# cytoKernel: robust kernel embeddings for assessing differential expression of single-cell data

**DOI:** 10.1093/bioinformatics/btaf399

**Published:** 2025-07-14

**Authors:** Tusharkanti Ghosh, Ryan M Baxter, Souvik Seal, Victor G Lui, Pratyaydipta Rudra, Thao Vu, Elena W Y Hsieh, Debashis Ghosh

**Affiliations:** Department of Biostatistics & Informatics, Colorado School of Public Health, University of Colorado, Anschutz Medical Campus, Aurora, CO 80045, United States; Department of Immunology and Microbiology, University of Colorado Anschutz Medical Campus, Aurora, CO 80045, United States; Department of Public Health Sciences, Medical University of South Carolina, Charleston, SC 29425, United States; Center for Translational Immunology, Benaroya Research Institute at Virginia Mason, Seattle, WA 98101, United States; Department of Statistics, Oklahoma State University, Stillwater, OK 74078, United States; Department of Biostatistics & Informatics, Colorado School of Public Health, University of Colorado, Anschutz Medical Campus, Aurora, CO 80045, United States; Department of Immunology and Microbiology, University of Colorado Anschutz Medical Campus, Aurora, CO 80045, United States; Department of Biostatistics & Informatics, Colorado School of Public Health, University of Colorado, Anschutz Medical Campus, Aurora, CO 80045, United States

## Abstract

**Motivation:**

High-throughput sequencing of single-cell data can be used to rigorously evaluate cell specification and enable intricate variations between groups or conditions to be identified. Many popular existing methods for differential expression target differences in aggregate measurement (mean, median, sum) and limit their approaches to detect only global differential changes.

**Results:**

We present a robust method for differential expression of single-cell data using a kernel-based score test, cytoKernel. CytoKernel is specifically designed to assess the differential expression of single-cell RNA sequencing and high-dimensional flow or mass cytometry data using the full probability distribution pattern. cytoKernel is based on kernel embeddings which employs the probability distributions of the single-cell data, by calculating the pairwise divergence/distance between distributions of subjects. It can detect both patterns involving changes in the aggregate, as well as more elusive variations that are often overlooked due to the multimodal characteristics of single-cell data. We performed extensive benchmarks across both simulated and real data sets from mass cytometry data and single-cell RNA sequencing. The cytoKernel procedure effectively controls the false discovery rate and shows favorable performance compared to existing methods. The method is able to identify more differential patterns than existing approaches. We apply cytoKernel to assess gene expression and protein marker expression differences from cell subpopulations in various publicly available single-cell RNAseq and mass cytometry datasets.

**Availability and implementation:**

The methods described in this paper are implemented in the open-source R package cytoKernel, which is freely available from Bioconductor at http://bioconductor.org/packages/cytoKernel.

## 1 Introduction

Technological advancements have revolutionized the field of high-throughput single-cell sequencing (sc-seq) (Tiberi *et al.* 2023, Wen *et al.* 2022), particularly single-cell RNA sequencing (scRNA-seq) (Saliba *et al.* 2014) and high-dimensional cytometry (flow or mass) (Pyne *et al.* 2009). sc-seq data have enabled in-depth exploration of biological processes at the single-cell level (Papalexi and Satija 2018, Stuart and Satija 2019). The sc-seq approach exceeds the capabilities of conventional bulk analysis by providing spatial-temporal insights into biological processes with high resolution (Chen 2022). Sc-seq plays a pivotal role in elucidating cellular heterogeneity, detecting rare cell subpopulations, isolating targeted biomarkers, and profiling distinctive molecular characteristics at the single-cell level (Giladi and Amit 2018, Ren *et al.* 2018).

A widely adopted approach in sc-seq for unraveling both intrinsic and extrinsic biological processes involves identifying genes (for scRNA-seq) or proteins (for high-dimensional cytometry) that exhibit differential expression (DE) (Zhang and Guo 2022). DE method facilitate the isolation and detailed examination of specific signals emanating from a particular cell subpopulation of interest. However, challenges arise due to the high heterogeneity and prevalence of zero counts in sc-seq data, complicating the statistical modeling and analysis (Luecken and Theis 2019, Kharchenko 2021).

Classical methods for analyzing DE, traditionally applied to bulk RNAseq data, have also been adapted for single-cell measurements, as indicated in studies by Soneson and Robinson (2018), Wang *et al.* (2019), Chen *et al.* (2020), and Squair *et al.* (2021). These methods have been shown to be effective on aggregated data by summarizing single-cell sequences into aggregate counts, also referred to as pseudo-bulk (PB) approach (Crowell *et al.* 2020), thus allowing the application of bulk RNAseq analysis methods. Traditional DE methods, particularly those based on PB data analysis, often focus on aggregating single-cell data to represent a “bulk” sample for each subject, thereby potentially overlooking intricate inter-subject variations in gene expression. In response to these complexities, several methods have been specifically developed for DE analysis in the context of single-cell data (Tiberi *et al.* 2023, You *et al.* 2023). These include scDD (Korthauer *et al.* 2016), SCDE (Kharchenko *et al.* 2014), BASiCS (Vallejos *et al.* 2015, Eling *et al.* 2018), and mixed models (MM) (Wills *et al.* 2013, Tung *et al.* 2017). Mixed-effects models, like the hurdle model described by Finak *et al.* (2015) and analyzed by Velmeshev *et al.* (2019) in autism research, are also commonly used. The hurdle model in MAST can be fit using mixed effects (Finak *et al.* 2015). The model incorporates fixed effects for variables like case/control status and random effects at the subject level which specifies a logistic regression model for the expression rate and a linear model for the logarithmic non-zero expression. Despite these innovations, these methods exhibit certain limitations. For example, BASiCS does not cater to cell-type-specific differential testing between conditions, scDD struggles with covariates and biological replicates, and others like PB, SCDE, MAST, and MM have shown limited efficacy in detecting differential patterns beyond mean differences in previous studies (Korthauer *et al.* 2016, Crowell *et al.* 2020). Conducting reliable statistical inference with MAST, such as testing for DE at a specified level of significance, within the framework of a fitted mixed-effect model, presents notable challenges. As detailed in (Zhang and Guo 2022), the incorporation of random effects often makes conventional likelihood ratio tests unsuitable in these contexts. Furthermore, the practical application of the hurdle model is constrained by its parametric assumptions, which might not be representative of real single-cell datasets.

To bridge this analytical gap, we introduce cytoKernel, a methodology for generating robust kernel embeddings via a Hilbert Space approach designed for sc-seq data analysis. Kernel-based methods have gained considerable significance in sc-seq data analysis for their ability to capture complex patterns and relationships that simpler aggregate-based approaches often fail to model (Wang *et al.* 2017, Bektaş and Gönen 2021, Ozier-Lafontaine *et al.* 2024). For example, PrognosiT illustrates the power of multiple kernel learning by predicting tumor volume from gene expression data bektas2021prognosit, while Zhang *et al.* (2022), Ozier-Lafontaine *et al.* (2024) introduced a kernel-based approach for single-cell DE analysis, although focused mainly on scRNA-seq, to identify distributional differences between groups. In contrast, cytoKernel leverages Jensen-Shannon divergence (JSD) and Reproducing Kernel Hilbert Spaces (RKHS) to detect differential patterns in both scRNA-seq and mass cytometry data, thus encompassing a wider range of single-cell omics. The symmetric and bounded properties of JSD make it well suited to capture multimodal or skewed distributions common in single-cell measurements. Unlike clustering-focused methods such as SIMLR (Wang *et al.* 2017), which aim to identify different cell types, cytoKernel is designed explicitly for DE analysis between predefined groups. CytoKernel diverges from traditional methods by conceptualizing the cell-type-specific gene expression of each subject as a probability distribution, rather than as a mere aggregation of single-cell data into PB measures. This probabilistic approach allows for a more sophisticated and nuanced comparison of gene expression across subjects, capturing complexities that PB methods may miss, such as subtle shifts and patterns in expression not observable through average measures alone.

## 2 Materials and methods

### 2.1 Overview of the semiparametric logistic regression model via functional hilbert space

#### 2.1.1 cytoKernel-PB: a pseudo-bulk approach

Traditional PB-based DE analysis usually involves assessing individual characteristics within cell subpopulations through a parametric model (Robinson *et al.* 2010, Love *et al.* 2014). This approach includes analyzing each feature by aggregating cells from each subject which generate a *P*-value. Then a feature is declared significant if its *P*-value is lower than a particular threshold, which is often corrected for multiple tests (Benjamini and Hochberg 1995, Benjamini and Yekutieli 2001).

For a fixed number of cells within a subject *i*, we observe the data triplet $\left\{z_{i},x_{i},y_{i}\right\}$, i=1,…,n where *n* is the total number of subjects obtained from a case–control study for the gene *g* (omitting the index g=1,…,G for simplicity). For subject *i*, $y_{i}$ is the case–control label taking values either 0 (control) or 1 (case), $x_{i}$ is a q×1 vector of covariates, $z_{i}$ is a $c_{i}\times 1$ single-cell vector. $c_{i}$ denotes the number of cells at single-cell resolution for subject *i*. In other words, the dimension of $z_{i}$ is equal to the size of the single-cell vector for the subject *i*.

A key element for the PB approach is the reduction of the single-cell vector $z_{i}$, which varies in dimension according to the single-cell count $c_{i}$ for each subject *i*, into a scalar quantity $z_{i}^{*}$. This reduction is a critical step in aggregating single-cell data into a PB format for the data. We can achieve this using various aggregation techniques, such as averaging, median calculation, or data pooling (Crowell *et al.* 2020). These aggregation methods are carefully selected to balance data simplification with the preservation of crucial biological signals (Finak *et al.* 2015).

Continuing with the development of the model for the PB approach, we assume that an intercept is included in $x_{i}$. The binary outcome $y_{i}$ depends on $x_{i}$ and $z_{i}^{*}$ through the following semiparametric logistic regression model:

(1) $$\text{logit}[P(y_{i}=1|x_{i},\;z_{i}^{*})]=x_{i}{}^{T}\beta +h(z_{i}^{*}),$$

where β is a q×1 vector of regression coefficients, and $h(z_{i}^{*})$ is an unknown cantered smooth function.

Model (1) is semiparametric because while β is finite-dimensional, we do not place any assumptions on h(·) except that it is assumed to lie in a certain functional space $H_{k}$. The covariate effects are modeled parametrically, while the PB scalar quantity $z_{i}^{*}$ is modeled nonparametrically. A nonparametric assumption for h(·) reflects our limited knowledge about the form of the genetic effect. Note that, when h(·)=0, $z_{i}^{*}$ has no association with the group labels $y_{i}$. Hence, a differentially expressed feature will lead to a rejection of the null hypothesis h(·)=0. Note, if $h(z^{*})=\gamma_{1}z^{*}$, for any arbitrary $\gamma_{1}$, model (1) becomes the generalized linear model (Goeman *et al.* 2011).

#### 2.1.2 cytoKernel-sc: a comprehensive single-cell approach capturing full distributional characteristics

For the sake of simplicity, we use the same notation from the previous section to describe the data triplets $\left\{z_{i},x_{i},y_{i}\right\}$, i=1,…,n where *n* is the total number of subjects obtained from a case–control study for gene *g*. The following model is considered for the analysis of DE of each feature in the cytoKernel methodology:

(2) $$\text{logit}[P(y_{i}=1|x_{i},\;z_{i})]=x_{i}{}^{T}\beta +h(z_{i}),$$

where h(·) is a centered smooth function in a RKHS (reproducing kernel Hilbert Space; Wahba (1990)) spanned by kernel *k* and $z_{i}$ is the single-cell vector with dimension $c_{i}\times 1$. Note, (2) is assumed to lie in $H_{k}$, the Hilbert Space. Such kernel-based models are more robust to model misspecification. Similar to the hypothesis in the cytoKernel-PB section, a differentially expressed gene will lead to the rejection of the null hypothesis $H_{0}$ : h(·)=0.

### 2.2 Kernel-based score test

We provide a detailed implementation of the kernel-based score test for sc-seq datasets, particularly focusing on the cytoKernel-PB version. The key adaptation for the cytoKernel-sc version involves substituting the variable $z_{i}^{*}$ of the cytoKernel-PB version with $z_{i}$ in the kernel-based score test framework. Apart from this specific modification, all other steps in the procedure remain consistent with the kernel-based semiparametric logistic regression model (Liu *et al.* 2008) for cytoKernel-sc. This approach upholds the methodological strictness of the kernel-based score test.

The RKHS $H_{k}$, is generated by a positive definite kernel function *k*. The statistical properties of $H_{k}$ imply that any function h(z) can be written as a linear combination of a given function k(·,·). Let *K* be the Gram matrix n×n with $K_{ij}\equiv k_{\rho }(z_{i}^{*},z_{j}^{*})$ being the reproducing kernel of the RKHS that contains h(·), and ρ be an unknown kernel parameter.

Assuming that h(·) lies within an RKHS, $h(\cdot )\in H_{k}$, β and h(·) can be simultaneously estimated by maximizing the penalized log-likelihood function

(3) $$\begin{array}{c} \ell [\beta ,h(\cdot )]=\sum_{i=1}^{n}[y_{i}\;\text{log}(\frac{\mu_{i}}{1-\mu_{i}})+\log(1-\mu_{i})]-\frac{\lambda }{2}\|h{\|}_{H_{k}}^{2}\\ =\sum_{i=1}^{n}[y_{i}(x_{i}{}^{T}\beta +h(z_{i}^{*}))-\\ \;\text{log}(1+\text{exp}\;\{x_{i}{}^{T}\beta +h(z_{i}^{*}\})]-\frac{\lambda }{2}\|h{\|}_{H_{k}}^{2}, \end{array}$$

where $\mu_{i}=P(y_{i}=1|x_{i},\;z_{i}^{*})$ and λ is a regularization parameter that balances between model complexity and goodness of fit (Liu *et al.* 2008). If λ=0, it reflects a saturated model at its boundaries, whereas λ=0 reduces the model to a fully parametric logistic regression model. There are two unknown parameters in ℓ[β,h(·)], the regularization parameter λ and bandwidth parameter ρ. We control the magnitude of the unknown function h(·) using λ. Meanwhile, ρ controls the smoothness of h(·) (Liu *et al.* 2008). The choice of ρ has a strong influence on the resulting estimate, so choosing an optimal value of ρ is critical.

According to Liu *et al.* (2008), it is possible to approach ℓ[β,k(·)] from a generalized linear MM (LMMs) perspective. The kernel estimator within the semiparametric logistic regression model is identical to the penalized quasi-likelihood function of a mixed logistic model, letting τ=1/λ denote the regularization parameter and ρ the bandwidth parameter (Liu *et al.* 2008, Zhan *et al.* 2015, Jensen *et al.* 2019). These parameters can be treated as variance components, where k(·)∼N(0,τK(ρ)) can be treated as a subject-specific random effect, and the covariance matrix K(ρ) is an n×n kernel matrix (Liu *et al.* 2008, Carpenter *et al.* 2021). This means that estimating β and k(·) can be done by maximizing the penalized log likelihood.

(4) $$\begin{array}{c} \ell [\beta ,h(\cdot )]=\sum_{i=1}^{n}[y_{i}\;\text{log}(\frac{\mu_{i}}{1-\mu_{i}})+\log(1-\mu_{i})]-\frac{\lambda }{2}\|h{\|}_{H_{k}}^{2}\\ =\sum_{i=1}^{n}[y_{i}(x_{i}{}^{T}\beta +h(z_{i}))\\ \;\text{log}(1+\text{exp}\;\{x_{i}{}^{T}\beta +h(z_{i}^{*}\})]-\frac{1}{2\tau }h^{T}Kh, \end{array}$$

where h=Kα and τ=1/λ (Liu *et al.* 2008). This provides an advantage because it allows for testing the null hypothesis $H_{0}:\tau =1/\lambda =0$ without explicit specification of the basis functions. The function h(·) can then be interpreted as subject-specific random effects with mean 0 and variance $\tau K_{\rho }$. Testing for an association between binary outcome and the distribution of features is then equivalent to testing the null hypothesis

(5) $$H_{0}:\tau =0\;\text{vs}\;H_{1}:\tau >0.$$

We use the modified kernel association test from Chen *et al.* (2016), adapted for small sample sizes, which is frequently applied in various gene expression and microbiome analyses, including metabolomics studies. The standard quadratic score statistic for kernel association tests is given by:

(6) $$Q(\beta ,\sigma ,\rho )=\frac{1}{\sigma^{2}}{\left(y-X\beta \right)}^{T}K(y-X\beta ),$$

where $y={\left(y_{1},y_{2},\ldots ,y_{n}\right)}^{T}$ and $X={\left(x_{1},x_{2},\ldots ,x_{n}\right)}^{T}$. To account for the high variability in the estimates of $\sigma^{2}$ with smaller sample sizes, adjustments are made. The null distribution of *Q* is then approximated as a weighted sum of $\chi^{2}$ distributions using the Davies method (Davies 1980).

#### 2.2.1 Gaussian kernel and empirical choice of the tuning bandwidth parameter

The Gaussian kernel is particularly well suited for modeling smooth, continuous variations in gene expression, subtle changes frequently encountered in single-cell data (Zhan *et al.* 2015, Carpenter *et al.* 2021, Seal *et al.* 2022). Unlike kernels with more rigid assumptions or mean-based approaches that may overlook nuanced patterns, the flexibility of the Gaussian kernel ensures that cytoKernel can capture these subtle shifts (Sriperumbudur *et al.* 2010). Furthermore, the Gaussian kernel is widely recognized for its strong theoretical properties and effectiveness in high-dimensional spaces, making it a robust choice for analyzing complex single-cell datasets.

Let $(z_{i}^{*},z_{j}^{*})\in \chi^{1}$ be two arbitrary PB gene expression measurements, where $\chi^{1}\equiv R$. We define a distance-based Gaussian kernel based on cytoKernel-PB:

(7) $$k_{\rho }(z_{i}^{*},z_{j}^{*})=\text{exp}[-\frac{d{\left(z_{i}^{*},z_{j}^{*}\right)}^{2}}{\rho }];\;(z_{i}^{*},z_{j}^{*})\in \chi^{1},$$

where $d(z_{i}^{*},z_{j}^{*})$ denotes the distance metric (square root of the Euclidean ($L_{2}$) norm) between pairwise PB gene expression measurements for subjects, *i* and *j*, respectively, and ρ>0. The Gaussian kernel is used, and the median of pairwise Euclidean distances between all $z_{i}^{*}$ and $z_{j}^{*}$ serves as an empirical estimate for the bandwidth parameter ρ. The selection of the Gaussian kernel is driven by its characteristic property, ensuring that the embedding of probability measures through the kernel function yields unique representations. Equation (7) is a well-defined kernel, as shown in Zhan, Patterson and Ghosh (2015).

### 2.3 Employing divergences in differential expression

To fully incorporate probability distributions, we propose a novel distance metric between subjects based on each gene *g* in the cytoKernel-sc approach. This metric is objective and can be easily tested for DE within a semiparametric logistic regression framework. We first discuss the concept of divergence or distance between two probability distributions, followed by its implementation.

#### 2.3.1 Embedding of conditionally negative definite via Hilbert Space

Consider a measure space (X,A,μ), where X is the sample space and A the σ-algebra of measurable subsets, with μ representing a dominating measure. In this space, we can define the set of all probability distributions P, where each distribution *P* maps elements of A to the interval X. Central to this is the JSD, a divergence measure $D_{JS}(\cdot ,\cdot )$ between two probability distributions $P_{1},P_{2}\in P$, defined as:

(8) $$\begin{array}{c} D_{JS}(P_{1},P_{2})=\int_{X}p_{1}(x)\;\text{log}\;\frac{2p_{1}(x)}{p_{1}(x)+p_{2}(x)}d\mu (x)+\\ \int_{X}p_{2}(x)\;\text{log}\;\frac{2p_{2}(x)}{p_{1}(x)+p_{2}(x)}d\mu (x), \end{array}$$

where $p_{1}$ and $p_{2}$ are the respective Radon-Nikodym derivatives of $P_{1}$ and $P_{2}$ with respect to μ.

In addition, the square root of the JS divergence, denoted here by $\sqrt{D_{JS}(\cdot ,\cdot )}$, enjoys the properties of a true distance metric, d((·,·): identity, symmetry, and triangle inequality (proof in the Supplementary Materials, available as supplementary data at *Bioinformatics* online). These properties enable JS divergence to effectively quantify the similarity between probability distributions, with smaller JS divergence values indicating higher similarity.

To maintain clarity, we use the same notation used in describing the data triplets $\left\{z_{i},x_{i},y_{i}\right\}$, i=1,…,n, where *n* is the total number of subjects obtained from a case–control study for the characteristic *j* (omitting the gene index *g*). Without loss of generality, for each subject *i*, the expression of the observed data from the single-cell vector $z_{i}$ is conceptualized as a continuous random variable, symbolized by $Z_{i}$ following the single-cell data framework for the construction of JS divergence-based distance metric (Seal *et al.* 2022). Now, $Z_{i}$ is observed across $n_{i}$ cells for subject *i*, represented as $Z_{1}^{i},Z_{2}^{i},\ldots ,Z_{n_{i}}^{i}$. The cumulative distribution function and the probability density function of $Z_{i}$ are represented by $F_{i}$ and $f_{i}$, respectively.

For the sake of completeness, let P be a convex set of probability measures defined on a measure space (X,A,μ), where X is the input sample space, and A the σ-algebra of measurable subsets, with μ representing a dominating measure. Within this setup, the collection P contains the probability distribution function $F_{i}$ for i=1,2,…,n.

The dissimilarity between two subjects *i* and $i^{\prime }$ in terms of the single-cell distribution is quantified using a distance measure, denoted $d(F_{i},F_{i^{\prime }})=\sqrt{D_{JS}(F_{i},F_{i^{\prime }})}$, based on the following equation:

(9) $$\begin{array}{c} d(F_{i},F_{i^{\prime }})=[\int_{0}^{1}f_{i}(x)\;\text{log}\;\frac{2f_{i}(x)}{f_{i}(x)+f_{i^{\prime }}(x)}dx+\\ \int_{0}^{1}f_{i^{\prime }}(x)\;\text{log}\;\frac{2f_{i^{\prime }}(x)}{f_{i}(x)+f_{i^{\prime }}(x)}dx{]}^{1/2}. \end{array}$$

A high value of $d(F_{i},F_{i^{\prime }})$ suggests a significant variance in the distribution or density between pairwise subjects *i* and $i^{\prime }$, whereas a low value indicates similar distributions. Subsequently, a distance matrix between all the pairwise subjects, denoted as $D={\left[[d(F_{i},F_{i^{\prime }})]\right]}_{n\times n}$, can be constructed. In practical scenarios, the density function $f_{i}$ is not known a priori. Hence, we estimate it using kernel density estimation (KDE), denoted as $\hat{f_{i}}$, based on the observations $Z_{j}^{i}$’s for $j=1,\ldots ,n_{i}$. The KDE is expressed as:

(10) $${\hat{f}}_{i}(z)=\frac{1}{n_{i}}\sum_{j=1}^{n_{i}}w_{b}(z-Z_{j}^{i}),$$

where $w_{b}$ is a Gaussian kernel with a bandwidth parameter *h*, selected according to Silverman’s rule of thumb (Silverman 1981). Utilizing these KDEs, $d(F_{i},F_{i^{\prime }})$ is approximated as:

(11) $$d(F_{i},F_{i^{\prime }})=[\sum_{r=1}^{R}{\hat{f}}_{i}(x_{r})\;\text{log}\;\frac{2{\hat{f}}_{i}(x_{r})}{{\hat{f}}_{i}(x_{r})+{\hat{f}}_{i^{\prime }}(x_{r})}+{\sum_{r=1}^{R}{\hat{f}}_{i^{\prime }}(x_{r})\;\text{log}\;\frac{2{\hat{f}}_{i^{\prime }}(x_{r})}{{\hat{f}}_{i}(x_{r})+{\hat{f}}_{i^{\prime }}(x_{r})}]}^{1/2},$$

where $x_{r}$, r=1,…,R are grid points within the interval [0, 1]. In our simulations and empirical analyses, we observed that the estimates remain stable for sufficiently large values of *R*. We set *R* to 1024 and used evenly spaced grid points, ensuring that the estimated densities sum up to 1 through appropriate scaling.

In the Supplementary Materials, available as supplementary data at *Bioinformatics* online, we define the conditionally negative distance (CND) and show that, $\sqrt{D_{JS}(\cdot ,\cdot )}$ induces an embedding of the distributions in a real Hilbert Space. The JSD can be described as the dot product of two probability distributions in a real Hilbert Space. That is, $\sqrt{D_{JS}(F_{i},F_{i^{\prime }})}=||\phi (F_{i})-\phi (F_{i})||$, where ϕ is a function that maps the probability distributions in a Hilbert Space. A fundamental property used to prove the existence of the embedding is the notion of CND function. Further, has shown that JS Divergence is CND.

### 2.4 Overview of multivariate distance matrix regression

For large number of subjects, semiparametric kernel regression offers computational advantages by calculating *P*-values based on the asymptotic distribution of the test statistic. However, for studies with small to moderate subject sizes, distance-based pseudo F tests, also referred to as multivariate distance matrix regression (MDMR), is preferred. MDMR relies on the resampling procedure to determine *P*-values. In both semiparametric kernel regression and MDMR approaches, the distance matrix must be converted to a kernel matrix.

We now briefly review MDMR (McArdle and Anderson 2001, Reiss *et al.* 2010, Zapala and Schork 2012). Let $\left(X,\sqrt{D_{JS}}\right)$ be a semi-metric space and Z be a random object taking values in X. Suppose, we observe independent draws of single-cell vector $z_{i}$ for subject i=1,2,…,n and $D={\left(d_{ij}\right)}_{n\times n}$ denotes the sample dissimilarity (distance) matrix for subject pair (i,j), i,j=1,2,…,n such that, $\left(d_{ij}\right)$ can be intuitively written as,

(12) $$d_{ij}=\sqrt{D_{JS}(z_{i},z_{j})}\equiv \sqrt{D_{JS}(F_{i},F_{j})}$$

Define the double centered matrix following the notations used in the previous section, we write, G=HAH with H=I−E/n and $A=-D^{2}/2$, where *I* denote an n×n identity matrix and *E* an n×n matrix where each element is equal to 1. The matrix *H* is a projection matrix, known as the centering matrix (Mardia *et al.* 1979).

Following the notation used in the kernel-based regression, we define X as an n×q covariate matrix. Let $X^{*}$ be the set of variables augmenting the binary outcome variable of interest $y={\left(y_{1},y_{2},\ldots ,y_{n}\right)}^{T}$ with all the covariates X, i.e. $X^{*}=[y,X]$ which can be expressed as a n×(q+1) design matrix with corresponding projection matrix $H_{X^{*}}=X^{*}{\left(X^{*}{}^{T}X^{*}\right)}^{-1}X^{*}{}^{T}$. Here $H_{X^{*}}$ is the traditional hat matrix.

MDMR assesses the association between Z and $X^{*}$ via the positive definite kernel matrix *G*. A pseudo F test statistic, as introduced by McArdle and Anderson (2001), is then represented by:

(13) $$F_{\text{pseudo}}=\frac{\mathrm{tr}(H_{X^{*}}GH_{X^{*}})}{\mathrm{tr}[(I-H_{X^{*}})G(I-H_{X^{*}})]},$$

where statistical significance is evaluated using the permutation test (Tang *et al.* 2016, Zhang *et al.* 2022).

### 2.5 cytoKernel-psrF

In this section, we propose a new test statistic, $F_{\text{sqrt}}$, a square root of the pseudo-F statistic derived from McArdle and Anderson (2001), Li *et al.* (2009), and Tang *et al.* (2016). In single-cell studies, moderate to high correlations are observed between feature expression, i.e. gene expression for scRNA-seq and protein marker expression for mass cytometry. To account for this correlated structure of the response variable (feature expression) and to increase the power of the test, we define a new statistic following Shi *et al.* (2023).

(14) $$F_{\text{sqrt}}=\frac{\mathrm{tr}(H_{X^{*}}G^{1/2}H_{X^{*}})}{\mathrm{tr}[(I-H_{X^{*}})G^{1/2}(I-H_{X^{*}})]},$$

where the square root of a matrix *A* is denoted as *B*, satisfying $B=A^{1/2}$. Let ${\left\{\lambda_{i}\right\}}_{i=1}^{n}$ and ${\left\{v_{i}\right\}}_{i=1}^{n}$ be the eigenvalues and eigenfunctions, respectively, of $\tilde{G}=H_{X^{*}}GH_{X^{*}}$. We define ${\tilde{G}}^{1/2}=\left(v_{1},\ldots ,v_{n}\right)\mathrm{diag}\left(\lambda_{1}^{1/2},\ldots ,\lambda_{n}^{1/2}\right){\left(v_{1},\ldots ,v_{n}\right)}^{⊤}$. The numerators of $F_{\text{pseudo}}$ and $F_{\text{sqrt}}$ can be intuitively represented as $\sum_{i=1}^{n}\lambda_{i}v_{i}^{⊤}{\tilde{v}}_{i}/m$ and $\sum_{i=1}^{n}\lambda_{i}^{1/2}v_{i}^{⊤}{\tilde{v}}_{i}/m$, respectively, where $H_{X^{*}}$ has eigenvalues equal to 1 and eigenfunctions ${\left\{{\tilde{v}}_{i}\right\}}_{i=1}^{n}$. The square root method potentially enhances the test efficacy by increasing the weight of significant factors when the response variables exhibit moderate correlation (Shi *et al.* 2023). In the presence of covariates, we employ the conditional distribution of the residuals rather than augmenting the binary variable of interest and all the covariates.

For scenarios where confounding variables X exist, we adopt a strategy of resampling the residuals post-regression of y on X. The linear predictor in this regression model is represented as γX. For cases where y is binary, the computation of the residual is performed via logistic regression, and the determination of the *P*-value is achieved through the application of the parametric bootstrap technique, as detailed by Davison and Hinkley (1997). The process involves the following steps:

- Perform a regression of y on X, leading to the maximum likelihood estimation (MLE) of γ, denoted as $\hat{\gamma }$. Calculate the residuals
  (15) $$R=y-\frac{\;\text{exp}(\hat{\gamma }X)}{1+\text{exp}(\hat{X})}.$$
  From these residuals, construct the observed square root of the pseudo-F statistic ($F_{\mathrm{sqrt}}$).
- For each resampling iteration, generate $y^{*}$ from a Bernoulli distribution with the success probability $\text{exp}(\hat{\gamma }X)/[1+\text{exp}(\hat{X})]$. Conduct a regression of $y^{*}$ on X to obtain the MLE ${\hat{\gamma }}^{*}$ for γ. Calculate the permutation residuals
  (16) $$R^{*}=y^{*}-\frac{\;\text{exp}({\hat{\gamma }}^{*}X)}{1+\text{exp}({\hat{\gamma }}^{*}X)}$$
  and form the resampled square root of the pseudo-F statistic $F_{\mathrm{sqrt}}$ based on $R^{*}$.
- Determine the final *P*-value by calculating the proportion of resampled $F_{\mathrm{sqrt}}$ statistics that exceed the observed statistic using a hierarchical permutation test.

This resampling strategy effectively addresses the influence of confounder covariates and provides a robust approach to statistical analysis in the presence of binary variables and confounding factors.

To evaluate whether there are significant differences between groups, cytoKernel uses a hierarchical permutation procedure in which individual observations (i.e. single-cell measurements) are shuffled rather than entire samples. Although this strategy does not fully satisfy the exchangeability condition typically required by permutation tests, our empirical evaluations indicate that it still provides appropriate control of type I errors and false discoveries.

Formally, let $F_{\mathrm{sqrt}}^{\text{obs}}$ be the test statistic observed computed on the original, unshuffled data. We generate *P* distinct permutations by redistributing single-cell observations among groups while maintaining the original sample sizes. For the *P*-th permutation, denote the resulting test statistic as $F_{\mathrm{sqrt}}$. We obtain an empirical *P*-value as follows:

$$\tilde{p}=\frac{\sum_{p=1}^{P}I\left(F_{\mathrm{sqrt}}\geq F_{\mathrm{sqrt}}^{\text{obs}}\right)+1}{P+1},$$

where I(·) is the indicator function that returns 1 if its argument is true and 0 otherwise.

To detect very small *P*-values accurately, cytoKernel adapts the number of permutations performed in stages. By default, the algorithm starts with 100 permutations. If the resulting $\tilde{p}\leq 0.1,$ the number of permutations is increased to 500; if the new $\tilde{p}\leq 0.01,$ it is increased to 2000; and finally, if $\tilde{p}\leq 0.001,$ cytoKernel performs up to 10 000 permutations. Users can customize both the threshold values and the number of permutations for each stage to balance computational cost with the desired precision.

When the response variable y is continuous, we employ a linear regression framework in conjunction with the Freedman-Lane permutation strategy (Freedman and Lane 1983) to generate a valid null distribution. This approach tests the association between y and predictors X by examining how much the observed residual structure departs from what would be expected under random permutations. The procedure proceeds as follows.

1. **Initial regression:** Fit a linear model of y on X to obtain the maximum likelihood estimate (MLE) $\hat{\gamma }$. Compute the residuals
  $$R=y-\hat{\gamma }X.$$
  From these residuals, construct the observed square root of the pseudo-*F* statistic ($F_{\text{sqrt}}$).
2. **Resampling:** For each resampling iteration:
  - Randomly permute the elements of R, yielding $R^{*}$.
  - Form the permuted response vector.
    $$y^{*}=\hat{\gamma }X+R^{*}.$$
  - Regress $y^{*}$ on X to obtain a new MLE ${\hat{\gamma }}^{*}$ for γ, and calculate the residuals.
    $$R_{\text{reg}}^{*}=y^{*}-{\hat{\gamma }}^{*}X.$$
  - Compute the resampled square root of the pseudo-*F* statistic $F_{\text{sqrt}}$ based on $R_{\text{reg}}^{*}$.
3. **Significance assessment:** The final *P*-value is determined by the proportion of resampled $F_{\text{sqrt}}$ statistics that exceed the observed $F_{\text{sqrt}}$ using the hierarchical permutation test.

## 3 Results

**Figure 1.**
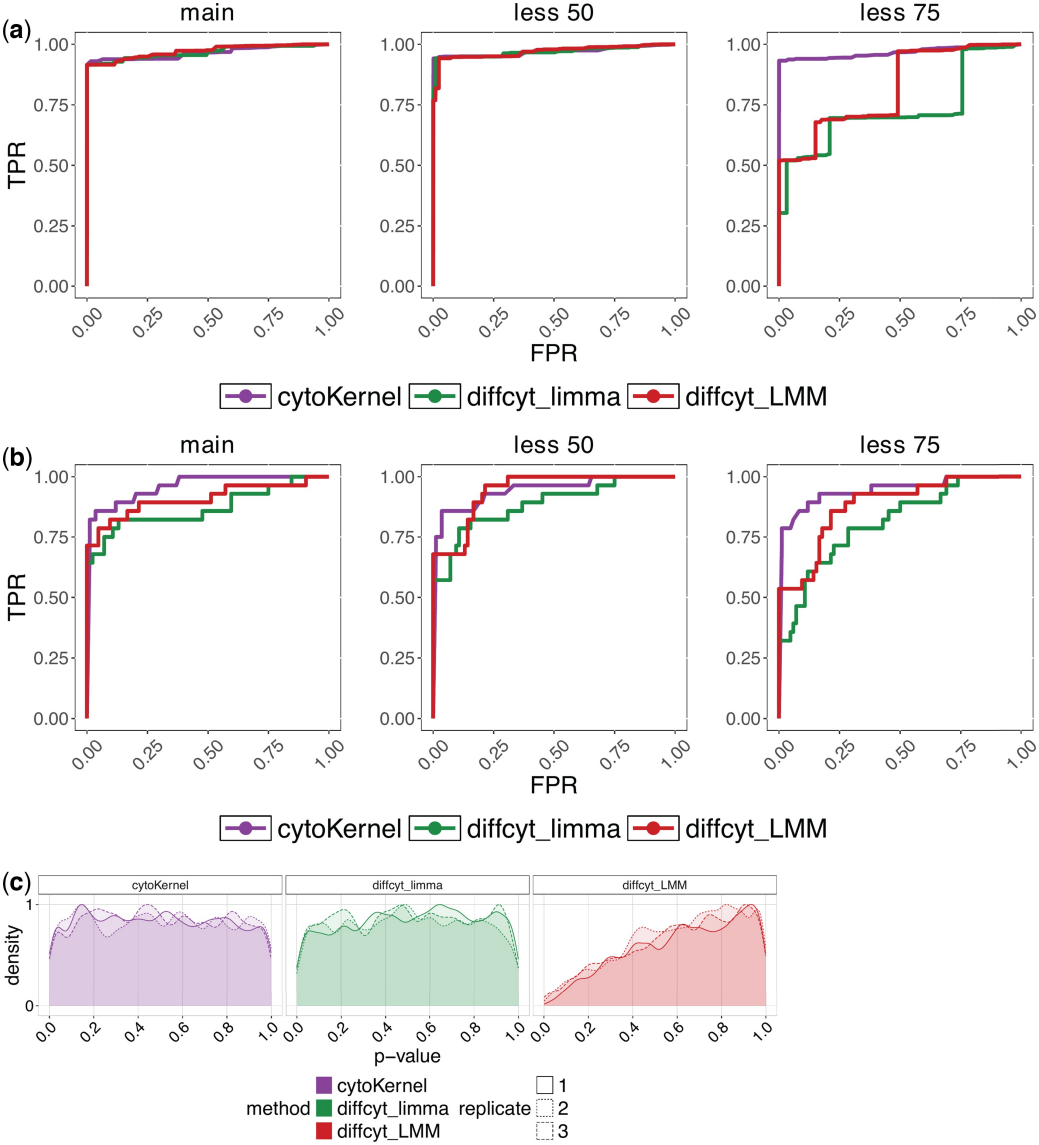
ROC curve in diffcyt non-null semi-simulated data. Each simulation consists of 88 435 cells and two groups of eight samples each. “main,” “less 50”, and “less 75” indicate the main simulation, and those where differential effects are diluted by 50% and 75%, respectively. We evaluated methods’ performance in terms of detecting DS for phosphorylated ribosomal protein S6 (pS6) in B cells, which is the strongest differential signal across the cell types in the dataset (Weber *et al.* 2019). Cells were clustered based on (a) manually annotated cell types and (b) unsupervised FLOWSOM clustering as in the distinct simulation study (Weber *et al.* 2019, Crowell *et al.* 2020, Tiberi *et al.* 2023). (c) Density of raw *P*-values in diffcyt null semisimulated data (Weber *et al.* 2019, Tiberi *et al.* 2023). Each replicate represents a different null simulation. Each replicate consists of 88 438 cells and two groups of eight samples each. Cells were clustered in an unsupervised manner (Weber *et al.* 2019).

**Figure 2.**
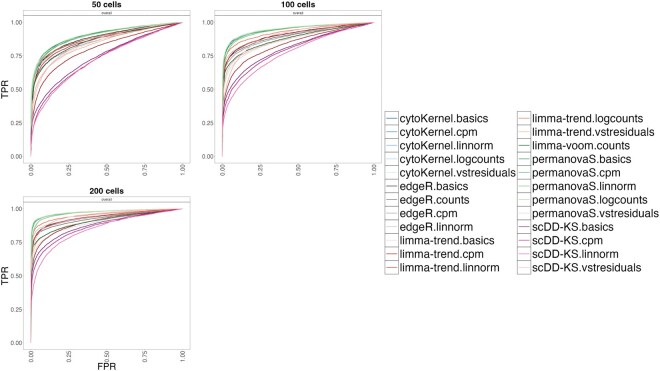
ROC curves generated using the distinct sensitivity (varying the number of cells) simulation design (Crowell *et al.* 2020, Tiberi *et al.* 2023). cytoKernel demonstrates better performance when varying the number of available cells. TPR versus FPR in muscat simulated data; with 50, 100, and 200 cells per cluster-sample combination, corresponding to a total of 900, 1800, and 3600 cells, respectively. Results are aggregated over the five replicate simulations of each differential type (DE, DP, DM, DB, and DV), contributing in equal fraction. Each individual simulation replicate consists of 4000 genes, three cell clusters and two groups of three samples each.

In our analysis of both simulated and real data, we focused on the cytoKernel-psrF variant of the cytoKernel method due to the small number of subjects in realistic sc-seq data settings. We will refer to cytoKernel-psrF as cytoKernel in the following simulations and real data sections. We presented a comparative analysis among three distinct cytoKernel methodologies: cytoKernel-PB, cytoKernel-sc, and cytoKernel-psrF in the context of the SplatPOP benchmarking. By varying the number of subjects: 20, 50, 100, we comprehensively assessed the performance of each method under different settings (Figs 1 and 2, available as supplementary data at *Bioinformatics* online).

### 3.1 Simulations

#### 3.1.1 Diffcyt

We analyzed the semi-simulated mass cytometry data from the designs implemented in Weber *et al.* (2019) and Tiberi *et al.* (2023). These simulations, which incorporated spike-in signals into experimental data (Bodenmiller *et al.* 2012), were designed to evaluate the performance of the cytoKernel method. This approach preserved real biological data characteristics while embedding a known ground truth. Our evaluation focused on cytoKernel and two diffcyt methods based on limma and LMMs, both of which previously showed superior performance on these datasets.

We examined three datasets from Weber and Soneson (2019): the primary DS dataset and two variants with 50% and 75% diluted differential effects (Weber *et al.* 2019). Each dataset contained 24 protein markers, 88, 435 cells, and two groups across eight samples each. We implemented two cell grouping approaches: one using eight manually annotated cell types (Fig. 1a) and another with 100 high-resolution clusters determined by unsupervised clustering method FLOWSOM (Van Gassen *et al.* 2015) (Fig. 1b).

**Figure 3.**
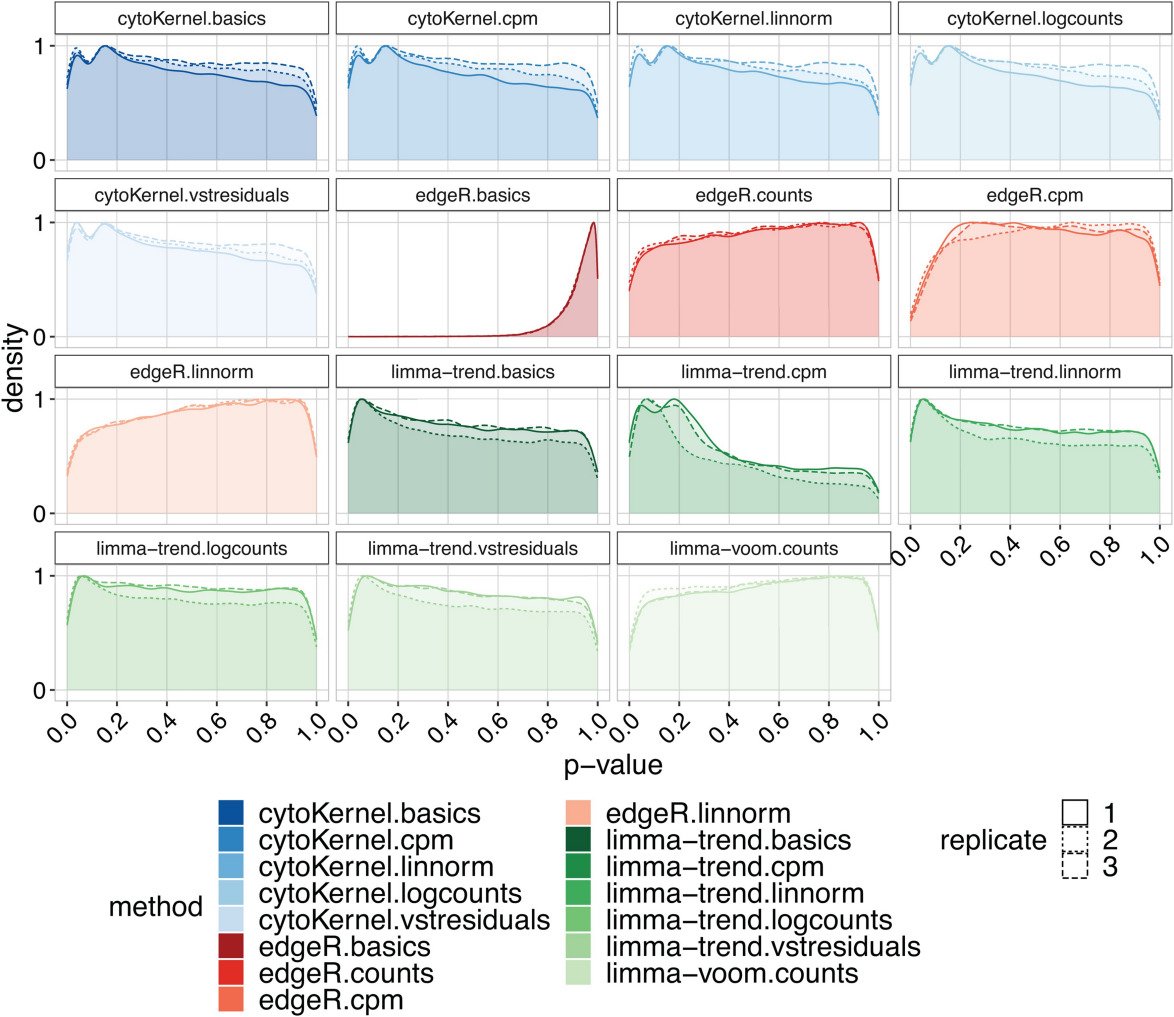
Density of raw *P*-values in null Kang Lupus data, comprising 11 854 cells across eight clusters. Each replicate in these datasets represents a random division of samples into two groups, highlighting the distribution of *P*-values obtained. The “cytoKernel” method demonstrates an almost-uniform distribution of null *P*-values.

**Figure 4.**
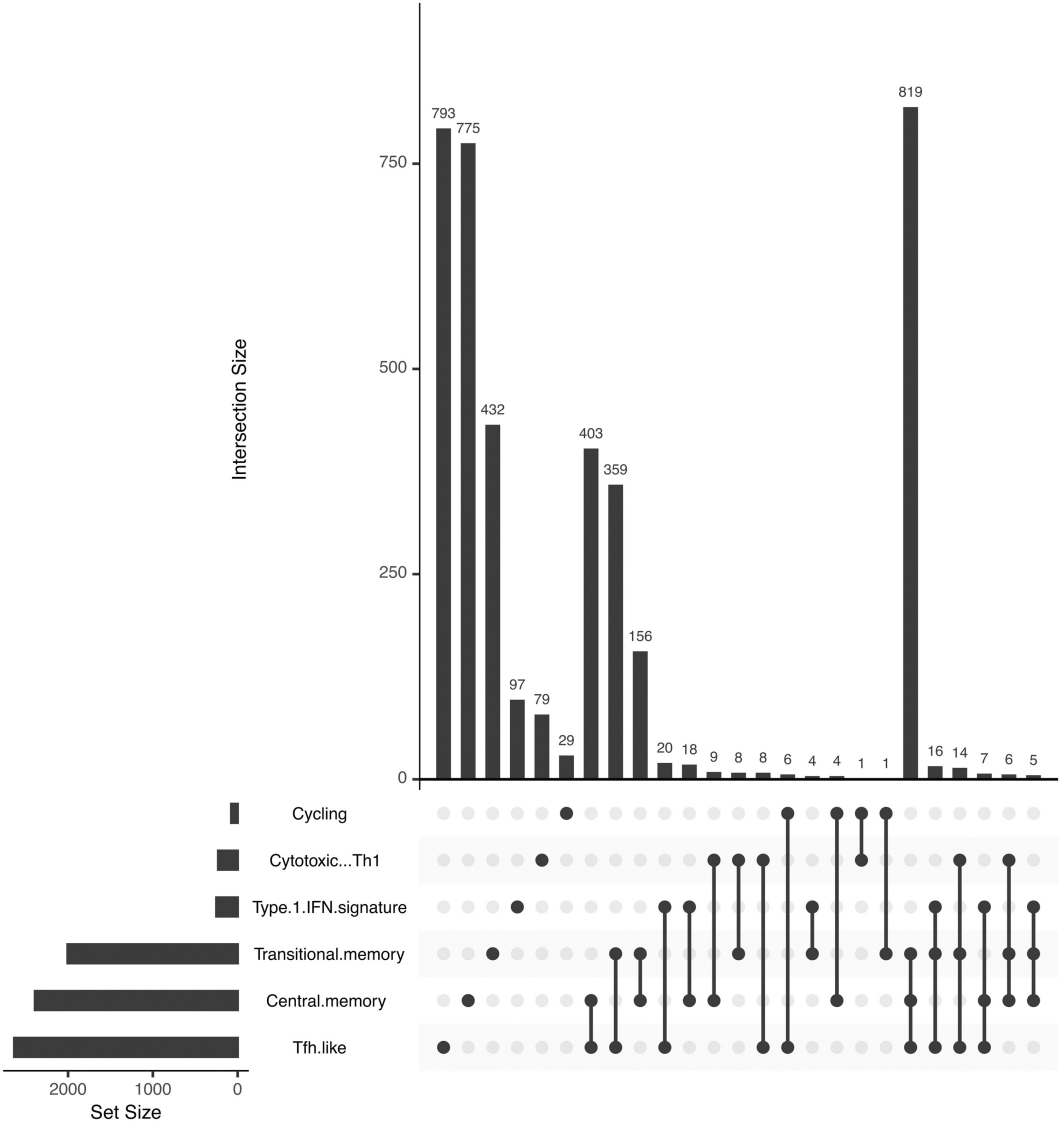
Analytical visualization of differential expression intersections across identified cell subpopulations. The graphical representation, generated via the UpSetR R package, compares the intersections of differential expression patterns identified by cytoKernel in the Bacher COVID-19 dataset. This comparison, focusing on mild-moderate versus severe subjects under an adjusted *P*-value criterion of <.1, highlights that the central memory and Tfh.like identify a greater number of differential expression patterns compared to Type 1-IFN-signature and Cytotoxic-Th1 subpopulations. Notably, the Cycling subpopulation emerges as the most conservative in identifying differential patterns. Furthermore, the memory subpopulations (Transitional memory and Central memory) display a notable consistency across Memory subpopulation sets.

The primary simulation study observed that the cytoKernel method exhibited a notably higher true positive rate (TPR) when cell-type labels were used. In contrast, when unsupervised clustering was applied, all methods showed comparable performance except 75% diluted differential effects where cytoKernel showed higher TPR (Fig. 1a). Notably, as the magnitude of the differential effect decreased, the disparity in performance became more pronounced. Specifically, the diffcyt methods showed a significant reduction in TPR, whereas cytoKernel not only maintained a higher TPR (Fig. 1b) but also effectively controlled the false discovery rate (FDR) (Figs 3 and 4, available as supplementary data at *Bioinformatics* online). This outcome indicates the robustness of cytoKernel in identifying even minor differential changes similar to distinct (Tiberi *et al.* 2023).

Furthermore, the study analyzed three replicated null datasets from Weber *et al.* (2019). These datasets consisted of 24 protein markers and 88, 438 cells distributed across eight cell types and were characterized by the absence of any differential effect. In these null scenarios, all evaluated methods, except for LMM, yielded *P*-values that were uniformly distributed, as shown in Fig. 1c. This uniform distribution of P-values signifies the reliability and validity of the cytoKernel method in scenarios free of differential effects.

#### 3.1.2 Muscat

**Figure 5.**
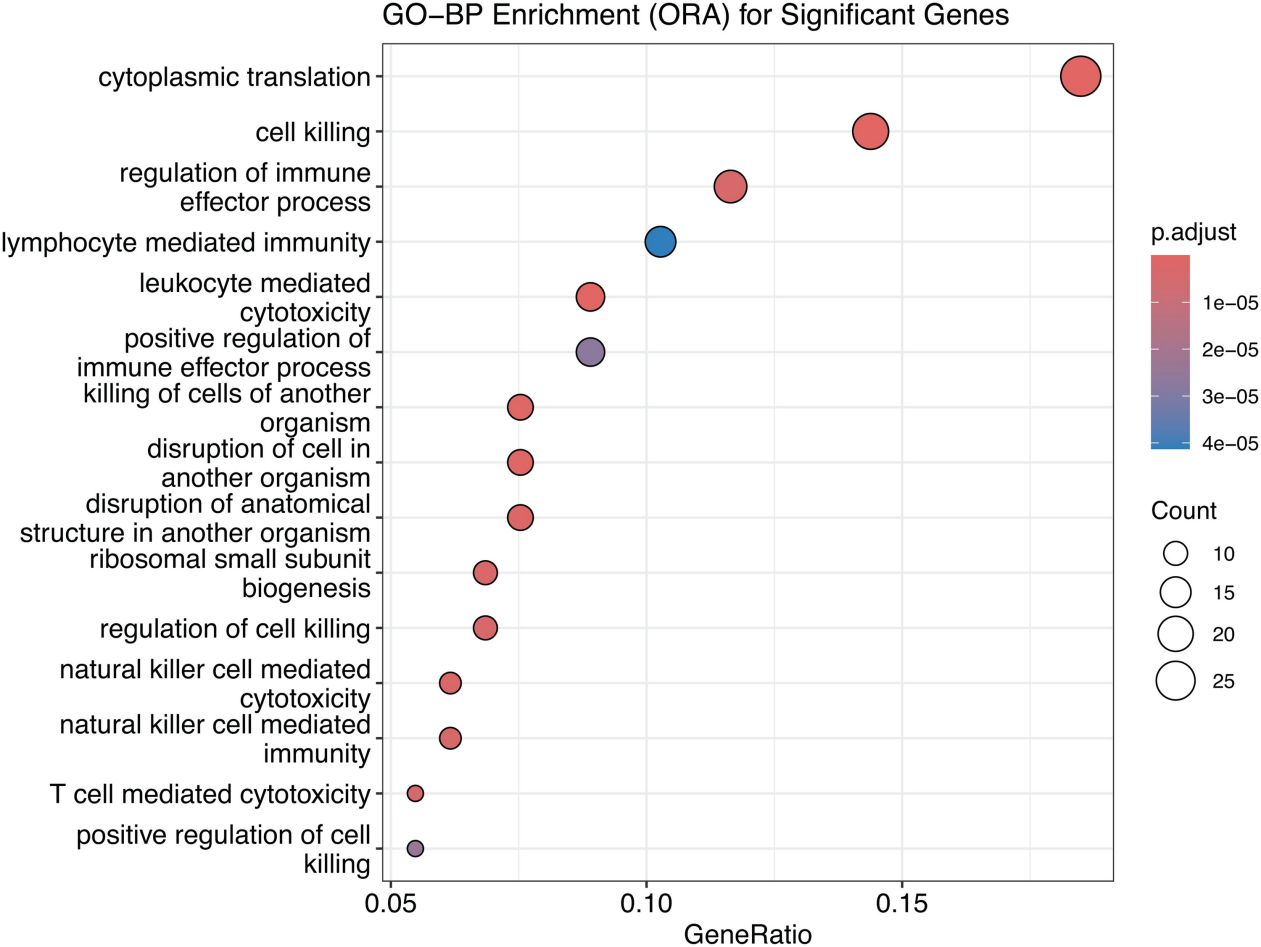
Dotplot visualizes top enriched GO-BP categories by adjusted *P*-value (color scale) and gene ratio (dotsize)Overrepresentation analysis (ORA) for the “Cytotoxic/Th1” cluster, extracted from the mild, moderate, and severe COVID-19 dataset (Bacher *et al.* 2020). Genes significantly associated with this cluster (adjusted P<.1) were mapped to Entrez IDs and analyzed using Gene Ontology Biological Process (GO-BP) terms.

We simulated droplet scRNA-seq data using muscat, as previously described in Crowell *et al.* (2020) and Tiberi *et al.* (2023). The study involved five replicates simulating differential characteristics profiles, with 10% of genes in each cluster exhibiting differential characteristics. These characteristics span across various differential patterns as described in Korthauer *et al.* (2016), Tiberi *et al.* (2023) (Fig. 5, available as supplementary data at *Bioinformatics* online):

- Differential expression (DE) indicating a shift in the entire distribution.
- Differential proportion (DP) indicating varied proportions in mixed distributions.
- Differential modality (DM) contrasting a single-mode with a dual-mode distribution.
- Differential modality and means (DB) comparing a single-mode and dual-mode distribution with equal means.
- Differential variability (DV), where two single-mode distributions with identical means but different variances were compared.

The simulations included 4000 genes across 3600 cells in three clusters, split into six groups with three subjects each, averaging 200 cells per subject per cluster. Our study examined how changing the number of cells per sample in each cluster influences the results. Specifically, we extended our simulations to include scenarios with 50, 100, 200, and 400 cells in each subject. This approach facilitated the evaluation of how variations in cell count influenced the sensitivity of the analysis. The results are compiled from five repeated simulations for each type of differential characteristic (DE, DP, DM, DB, and DV), with each type contributing an equal fraction. Further, we compared our simulation performance with IDEAS-PERMANOVA-S (Tang *et al.* 2016, Zhang *et al.* 2022).

The study examined six normalization techniques: counts, counts per million (CPMs), the logcounts method from scater (McCarthy *et al.* 2017), linnorm (Yip *et al.* 2017), BASiCS (Vallejos *et al.* 2015, Eling *et al.* 2018), and the residuals from variance stabilizing normalization (vstresiduals) (Hafemeister and Satija 2019). These methods were evaluated alongside Poisson-based (PB) procedures from muscat, such as edgeR (Robinson *et al.* 2010) and limma-trend (Ritchie *et al.* 2015). These PB methods have been recognized for their effectiveness in differential analysis of scRNA-seq data (Crowell *et al.* 2020, Skinnider *et al.* 2021, Squair *et al.* 2021). In addition, scDD (Korthauer *et al.* 2016)-based methods were assessed, as implemented in distinct (Tiberi *et al.* 2023). scDD utilizes a nonparametric method to detect distribution changes in scRNA-seq data, employing only the Kolmogorov–Smirnov test (scDD-KS). We omit the permutation approach (scDD-perm) due to its high computational cost.

CytoKernel maintained or surpassed the performance of leading methods such as edgeR.linnorm and limma-trend.logcounts in scenarios with 50, 100, and 200 cells as shown in the ROC curves (Fig. 2, Table 1). It demonstrated robust performance across all scenarios, effectively balancing statistical power with FDR control (Fig. 6, available as supplementary data at *Bioinformatics* online). Overall, increasing cell counts generally improved the performance of all methodologies, particularly in detecting differential effects, as evidenced by higher TPRs. Further analysis indicated that the performance of cytoKernel was minimally influenced by different normalization inputs, likely due to its inherent nonparametric design.

**Table 1.** Area under curve (AUC) values generated using the distinct sensitivity (varying the number of cells) simulation design (Crowell *et al.* 2020, Tiberi *et al.* 2023).

Method	AUC (50 cells)	AUC (100 cells)	AUC (200 cells)
permanovaS.basics	0.90	0.94	0.96
permanovaS.cpm	0.91	0.94	0.96
permanovaS.linnorm	0.92	0.95	0.97
permanovaS.logcounts	0.88	0.93	0.95
permanovaS.vstresiduals	0.90	0.94	0.96
cytoKernel.basics	0.91	0.95	0.97
cytoKernel.cpm	0.92	0.95	0.97
cytoKernel.linnorm	0.93	0.96	0.98
cytoKernel.logcounts	0.92	0.96	0.98
cytoKernel.vstresiduals	0.93	0.96	0.98
edgeR.basics	0.90	0.94	0.95
edgeR.counts	0.89	0.93	0.95
edgeR.cpm	0.92	0.95	0.97
edgeR.linnorm	0.93	0.96	0.98
limma-trend.basics	0.91	0.93	0.95
limma-trend.cpm	0.91	0.91	0.95
limma-trend.linnorm	0.92	0.91	0.98
limma-trend.logcounts	0.91	0.89	0.96
limma-trend.vstresiduals	0.91	0.93	0.92
limma-voom.counts	0.92	0.95	0.97
scDD-KS.basics	0.77	0.81	0.83
scDD-KS.cpm	0.76	0.80	0.82
scDD-KS.linnorm	0.78	0.82	0.84
scDD-KS.vstresiduals	0.79	0.83	0.85

cytoKernel demonstrates better performance when varying the number of available cells. TPR versus FPR in muscat simulated data; with 50, 100, and 200 cells per cluster-sample combination, corresponding to a total of 900, 1800, and 3600 cells, respectively. Results are aggregated over the five replicate simulations of each differential type (DE, DP, DM, DB, and DV), contributing in equal fraction. Each individual simulation replicate consists of 4000 genes, three cell clusters and two groups of three samples each.

CytoKernel ran in approximately 1.2–1.5 minutes per simulation, which is longer than PB methods (0.1–0.2 minutes) and scDD-KS (0.5–0.7 minutes), but still considerably quicker than IDEAS-PERMANOVA-S based approaches (permanovaS) (6.4–8.6 minutes) and scDD-perm (204.3–782.1 minutes) (Table 1, available as supplementary data at *Bioinformatics* online). All methods utilized 8 CPU cores except for PB approaches, which relied on a single core due to the absence of parallelization support.

We further extended our simulation study by incorporating a batch effect *cell-type-specific*, in which each cell type is subject to a different batch-induced change (Crowell *et al.* 2020, Tiberi *et al.* 2023). Specifically, we introduced two batches (*b*1, *b*2): one experimental group contained two samples from *b*1 and one from *b*2, while the other group contained two samples from *b*2 and one from *b*1. This design allowed us to assess the ability of each method to account for batch‐driven heterogeneity. The resulting differential analyzes remained largely consistent with our baseline findings (Fig. 7, available as supplementary data at *Bioinformatics* online), indicating that *cytoKernel* effectively mitigates confounding effects attributable to complex batch structures.

### 3.2 Real data

#### 3.2.1 Null experimental data

For evaluating FPRs using real data, two scRNA-seq datasets under identical experimental conditions were analyzed, as previously examined in Tiberi *et al.* (2023). These datasets were not expected to exhibit differential characteristics. The focus of the analysis was on cytoKernel and PB methods, considering the high computational requirements and limited effectiveness of MM methods (Crowell *et al.* 2020), as well as the elevated FDR noted in scDD models (Korthauer *et al.* 2016). The analysis included gene-cluster pairs with at least 20 non-zero cells in all subjects.

The first dataset, “Kang”, includes 10× droplet-based scRNA-seq data of peripheral blood mononuclear cells from eight Lupus patients, both before and after a 6-hour treatment with interferon-β (INF-β) (Kang *et al.* 2014). This dataset comprises 35 635 genes and 29 065 cells, manually categorized into eight cell types. Due to its outlier characteristics, one patient was excluded (Tiberi *et al.* 2023). The analysis concentrated on singlet cells and cells assigned to specific populations, considering only the control samples, which resulted in 11 854 cells and 10 891 genes. Three replicated datasets were then generated by randomly dividing the seven remaining control samples into two groups of three and four.

The second dataset, called “T cells,” is a Smart-seq2 scRNA-seq dataset encompassing 19 875 genes from 11 138 T cells obtained from the peripheral blood of 12 colorectal cancer patients (Zhang *et al.* 2019). Cells were sorted into 11 clusters utilizing igraph (Csardi and Nepusz 2006). To create replicated datasets, the 12 patients were randomly split into two groups of six, forming three replicates.

In the analyses of the “Kang” dataset under null conditions, it was observed that the application of limma-trend, particularly when utilizing CPMs, resulted in elevated FPRs. The *P*-values derived from cytoKernel indicated a slight increment toward zero. In contrast, edgeR and limma-voom demonstrated more conservative properties, thereby providing enhanced control over FPRs, as depicted in Fig. 3 and (Table 2, available as supplementary data at *Bioinformatics* online). The “T cells” dataset is presented in the Fig. 8, available as supplementary data at *Bioinformatics* online, Table 3, available as supplementary data at *Bioinformatics* online. With regard to normalization methods, both linnorm and BASiCS were found to generate the most conservative *P*-values, consequently leading to the lowest FPRs observed in the study.

#### 3.2.2 Bacher COVID-19 data

In a study conducted by Bacher *et al.* (2020), a detailed statistical analysis was performed to identify differentially expressed genes in the subpopulations of T cells of central memory and Type I interferon-gamma (IFNG) in patients with varying severity of COVID-19. In the study, the Benjamin–Yekatueli procedure (Benjamini and Yekutieli 2005) was used for the FDR control, specifically tailored for dependent gene sets. This statistical approach improves the accuracy of identifying differentially expressed genes by accounting for dependencies within the data. Upon examining the gene-cluster combinations flagged by cytoKernel using the criteria: adjusted *P*-value<.1, we identified several significant genes. UMAP visualizations effectively delineated these subpopulations in correlation with disease severity (mild-moderate and severe). The primary focus of the study was to identify differentially expressed genes from subpopulations of interest and to visually inspect the empirical distributions of crucial genes such as IL2 and IFNG (Fig. 9, available as supplementary data at *Bioinformatics* online). This approach aimed to uncover non-canonical DE characteristics associated with COVID-19 severity. Further, among the genes identified by cytoKernel (Supplementary Materials Additional File 1, available as supplementary data at *Bioinformatics* online), many are associated with immune response pathways dysregulated in severe COVID-19, such as interferon signaling and cytokine production (Bacher *et al.* 2020). For example, IFNG and IRF1 are key modulators of Th1 response and type I/II interferon pathways. Meanwhile, genes such as GZMB, PRF1, and NKG7 contribute to direct cytotoxicity, reflecting potent antiviral effector functions. The detection of IL18R1 and CD69, an early activation marker in T cells, further underscores active inflammatory signaling and T-cell activation and cytokine production function. In particular, less-canonical transcripts, including GBP5, APOBEC3G (a cytidine deaminase involved in viral restriction) and various ribosomal components (e.g. RPS27A, RPL36), highlight the multifaceted nature of antiviral responses in severe COVID-19 disease. These findings highlight the ability of cytoKernel to detect biologically significant patterns that traditional mean-based methods may overlook. Additionally, in Fig. 4, concordance plot using the UpSetR package provided insight into overlaps and divergences of gene expression in different groups of T cells, enhancing our understanding of immune responses to SARS-CoV-2. It details how DE patterns are compared across identified cell subpopulations, particularly between mild-moderate and severe COVID-19 cases, using a significance threshold of P<.1. The analysis reveals that central memory and Tfh-like subpopulations show a higher incidence of DE patterns compared to Type 1-IFN signature and Cytotoxic-Th1. Interestingly, the cycling subpopulation is characterized as the most conservative in terms of differential pattern identification. Additionally, a significant consistency in DE is noted across memory subpopulations, specifically transitional and central memory.

To further elucidate the functional programs underlying these significant gene sets, we performed an overrepresentation analysis using clusterProfiler, which identified terms enriched gene ontology (GO) 219 in the significantly upregulated genes (n=146 assigned to Entrez ID) (Fig. 5 and Fig. 10, available as supplementary data at *Bioinformatics* online). In particular, several top-ranked categories reflect robust cytotoxic and translational activities, including cytoplasmic translation (GO: 0002181), cell killing (GO: 0001906), and leukocyte-mediated cytotoxicity (GO: 0001909). This enrichment underscores the strong effector functionality in the Cytotoxic/Th1 cluster, where genes involved in T-cell-mediated killing, interferon signaling, and ribosomal machinery appear to be highly upregulated. This aligns with a Th1-driven immune response, supported by genes such as IFNG, PRF1, and GZMB, which play crucial roles in antiviral defense. The presence of terms such as killing cells of another organism (GO: 0031640) highlights the critical direct cytolytic capacity to control SARS-CoV-2 infection. Collectively, these enriched pathways provide a clearer portrait of how specific T-cell subpopulations could orchestrate potent and targeted immune responses, providing additional context for the interaction between memory cells, Tfh-like functions, and cytotoxic/Th1 phenotypes in COVID-19. Furthermore, cytoKernel helped identify subtle expression changes correlated with polyfunctionality, specifically capturing how low-avidity SARS-CoV-2-reactive CD4^+^ T cells differ from their high-avidity counterparts. By comparing mean expression, distributional patterns, and local gene expression densities, cytoKernel detected otherwise overlooked genes and cell states linked to low-avidity dysfunctional T-cell responses, reflecting the qualitative defects highlighted in Bacher *et al.* (2020). This refined resolution in identifying gene-cluster combinations complements both the overrepresentation findings and the broader immunological narrative: even when T-cell frequencies are high in severe disease, the underlying low-availability profile can undermine effective viral control. Thus, integrating the output of cytoKernel with analysis at the pathway level provides a more robust, multilayered understanding of the responses of SARS-CoV-2-specific T cells.

## 4 Discussion

Traditionally, sc-seq datasets have faced limitations due to high sequencing costs and technical constraints. This has resulted in data being collected from various cell types, but from only a few subjects. Consequently, the focus has been on identifying DE between cell types. There has been less emphasis on comparing multiple subjects between the case and control groups. However, with decreasing costs and improved accessibility of sc-seq datasets, there is a growing availability of vast case–control study datasets, particularly in complex human disease research. To address the needs of these emerging datasets, we have developed a novel method, cytoKernel, tailored for DE analysis in single-cell data. “cytoKernel” utilizes a robust multi-subject full-distribution kernel embeddings framework, designed to identify differential patterns between groups of distributions, especially effective in scenarios where mean changes are not evident.

Our method has demonstrated superior performance in extensive benchmarks against both simulated and experimental datasets from scRNA-seq and mass cytometry, offering better control over false-negative and false discovery rates and identifying more DE patterns than traditional methods. Notably, it shows higher statistical power than PB methods and other statistical frameworks, like Bayesian hierarchical framework-based scDD. “cytoKernel” also adeptly adjusts for sample-level, cell-cluster-specific covariates, including batch effects. The versatility of “cytoKernel” extends beyond identifying differential patterns, and its performance remains consistent across various normalization approaches. This method, therefore, marks a significant advancement in the analysis of high-throughput single-cell data, particularly in biomedical research involving complex disease groups.

In addition to revealing classical cytotoxic mediators such as IFNG, PRF1, and GZMB, the cytoKernel approach also captured subtle expression changes in genes not prominently featured in the original low-avidity CD4^+^ T-cell study (Bacher *et al.* 2020). For example, noncoding transcripts such as FO704657.1 and AL606491.1, along with less-characterized ribosomal components RPS27A, RPL22L1, and RPS29, were flagged as differentially expressed in specific groups, although they were not highlighted in the original low-avidity CD4^+^ T-cell study (Bacher *et al.* 2020). These genes may play an underappreciated role in shaping T-cell activation states, cellular metabolism, or translational reprogramming, potentially modulating the phenotype of low-avidity CD4^+^ T cells. CytoKernel could detect these more nuanced patterns, often overlooked by simpler threshold-based methods, thus bridging the gap between global significance metrics and biologically meaningful, yet subtler, transcriptional signals.

Furthermore, cytoKernel is robust to datasets with extreme sparsity and high zero inflation. By focusing on the full distribution of expression values rather than relying solely on mean or variance estimates, which zeros can skew, cytoKernel can detect differential patterns even in highly sparse data. In our implementation, DE analysis is performed separately for each predefined cell type or group, in accordance with standard practices in single-cell analysis. However, the method can also be adapted for pooled analysis if desired, providing users flexibility.

Data-driven methods, particularly those that identify DE, are vital in guiding experimental research focused on complex diseases such as Lupus and COVID-19. These approaches are essential in genetic research, where discerning a precise list of key genes is far more beneficial than compiling an extensive but less relevant gene catalog. The cytoKernel method exemplifies this utility with its nonparametric flexibility, strong adherence to controlling type I error rates, and ability to detect crucial differential patterns of genes. This makes it highly suitable for DE analyses in case–control studies with multiple subjects in each group. By efficiently isolating significant genes in these areas, cytoKernel provides a solid foundation for a deeper experimental investigation, improving our understanding of these complex disease conditions. Future extensions of cytoKernel include handling multiple groups using pairwise JSD comparisons or kernel analysis of variance and extending to continuous outcomes through kernel regression frameworks. These developments will broaden the applicability of cytoKernel to a wider range of experimental designs.

## Supplementary Material

btaf399_Supplementary_Data

## Data Availability

The cytoKernel R package is freely accessible through Bioconductor (https://bioconductor.org/packages/cytoKernel), with the development version available on GitHub (https://github.com/Ghoshlab/cytoKernel). The scripts used to run all analyses are available on Zenodo (DOI: 10.5281/zenodo.14003780).The diffcyt simulated dataset is accessible via both FlowRepository (accession ID: FR-FCM-ZYL8 [Weber *et al.* 2019]) and the HDCytoData package (Weber and Soneson 2019) on Bioconductor. The Kang dataset is available through the muscData Bioconductor package (Crowell *et al.* 2020), while the T-cell dataset is deposited in the European Genome-phenome Archive under accession ID EGAD00001003910 (Zhang *et al.* 2019). Additionally, Bacher COVID-19 data generated in this study can be accessed from the BacherTcellData scRNA-seq Bioconductor package.
