# Supplementary material for: cytoKernel: robust kernel embeddings for assessing differential expression of single-cell data
Source: Bioinformatics. 2025 Jul 14;41(7):btaf399. doi: 10.1093/bioinformatics/btaf399 (PMC12312792; doi:10.1093/bioinformatics/btaf399)
Supplement: btaf399_Supplementary_Data [file btaf399_supplementary_data.pdf]

Supplementary to "cytoKernel: Robust kernel embeddings for assessing differential expression of single cell data"

Tusharkanti Ghosh[1], Ryan M Baxter[2], Souvik Seal[3], Victor G Lui[4], Pratyaydipta Rudra[5], Thao Vu[1], Elena WY Hsieh[2], and Debashis Ghosh[1]

[1]Department of Biostatistics and Informatics, Colorado School of Public Health, University of Colorado Anschutz Medical Campus, Aurora, CO, USA

[2]Department of Immunology and Microbiology, University of Colorado Anschutz Medical Campus, Aurora, CO, USA

[3]Department of Public Health Sciences, Medical University of South Carolina, Charleston, SC, USA

[4]Center for Translational Immunology, Benaroya Research Institute at Virginia Mason, Seattle, WA, USA

[5]Department of Statistics, Oklahoma State University, Stillwater, OK, USA

Keywords: scRNAseq, Mass Cytometry, differential pattern, nonparametric methods

1 Accounting for overdisepersion

The SKAT statistic for binary outcomes, as originally conceived by Lee et al. (2012), is based on the assumption of no overdispersion. This is characterized by setting the dispersion parameter for the binomial distribution at $\phi = 1$. The statistic is defined as follows:

$$Q(\widehat{\boldsymbol{\beta}}, \rho) = (\mathbf{y} - \widehat{\boldsymbol{\mu}})^T \mathbf{K}_\rho (\mathbf{y} - \widehat{\boldsymbol{\mu}}) \tag{1}$$

In this Equation, $\widehat{\boldsymbol{\mu}}$ is derived from the logit inverse of $\widehat{\boldsymbol{\beta}}$, with $\widehat{\boldsymbol{\beta}}$ representing the estimate of $\boldsymbol{\beta}$ under the null hypothesis $H_0 : h(\cdot) = 0$ within the framework of equation (1). The function $h(\cdot)$ lies within the Reproducing Kernel Hilbert Space (RKHS) $\mathcal{H}_k$, generated by $k_\rho(\cdot, \cdot)$. It is important to note that under the null hypothesis $H_0 : h(\cdot) = 0$, the parameter ρ is not included in the model. Consequently, the test statistic ρ cannot be estimated under this null hypothesis, which affects the determination of significance levels. Further sections will delve into selecting an empirical estimate for ρ and the appropriate kernel.

The challenge posed by this non-standard testing problem was addressed by Davies (1980, 1987), who developed a score statistic conforming to a mixed chi-squared distribution. As sample size increases, this statistic tends to follow this distribution more closely, enhancing the accuracy of the asymptotic distribution (Duchesne and De Micheaux, 2010). However, in smaller samples, the variability in estimating σ^2 (also chi-squared distributed) introduces uncertainty. This can lead to imprecise approximations of the null distribution for the test statistic $Q(\widehat{\boldsymbol{\beta}}, \rho)$. Such imprecision often yields conservative results when employing the Davies method. It is crucial to recognize that this conservativeness arises not from the Davies method per se, but rather from the variability in estimating σ^2.

1.0.1 Approximate p-value computation

To address the issue of p value computation for kernel based score test with small sample sizes, (Chen et al., 2016) proposed an approximate test based on the SKAT score statistic for binary outcome, which resolves the small sample problem of the score statistic $Q(\widehat{\boldsymbol{\beta}}, \hat{\rho})$. The distribution of $Q(\tilde{\boldsymbol{\beta}}, \hat{\rho})$ satisfying the asymptotic properties is provided in the supplementary materials. We use an empirical estimate of ρ,i.e., $\hat{\hat{\rho}}$. Following Chen's method (Chen et al., 2016), we write a modified score statistic to account for potential overdispersion, which is generally seen in real data due to unmodelled risk factors in the link function.

$$Q(\widehat{\boldsymbol{\beta}}, \hat{\rho}) = \frac{1}{\hat{\phi}}(\mathbf{y} - \widehat{\boldsymbol{\mu}})^T \mathbf{K}_{\hat{\rho}}(\mathbf{y} - \widehat{\boldsymbol{\mu}}) \tag{2}$$

where $\hat{\nu} = \hat{\sigma}^2$, such that $\hat{\sigma}^2$ is the estimator of the dispersion parameter $\nu = \sigma^2$.

1.0.2 Estimation of ν

The estimation ν is based on the iteratively reweighted least squares (IRLS) algorithm for generalized linear models. The tth step of IRLS can be expressed as solving the linear null model:

$$\tilde{\mathbf{y}}^t = \mathbf{X}\boldsymbol{\beta} + \boldsymbol{\epsilon}, \tag{3}$$

where $\boldsymbol{\epsilon} \sim N(0, \sigma^2)$ and $\tilde{\mathbf{y}}^t$ is the working response for the estimation purpose, $\sigma^2 = \nu$ the dispersion parameter, and $\tilde{\mathbf{W}}^t$ is the diagonal weight matrix with elements $\tilde{W}^t_{ii} = \tilde{\mu}^t_i(1 - \tilde{\mu}^t_i)$ (Hastie and Pregibon, 2017). To denote the quantities of interest at convergence, the superscript t is dropped. Let $\mathbf{D}$ be a diagonal matrix with the ith diagonal element $\tilde{\mu}_i(1 - \tilde{\mu}_i)$. The score statistic for binary outcomes accounting for overdispersion can be written as:

$$Q(\tilde{\boldsymbol{\beta}}, \hat{\rho}) = \frac{1}{\hat{\sigma}^2}(\tilde{\mathbf{y}} - \tilde{\boldsymbol{\mu}})^T \mathbf{D}\mathbf{K}_{\hat{\rho}}\mathbf{D}(\tilde{\mathbf{y}} - \widehat{\boldsymbol{\mu}}) \tag{4}$$

$\hat{\sigma}^2$ is the dispersion estimate at convergence based on reweighted least squares. If we define $\tilde{\mathbf{y}}^* = \mathbf{W}^{1/2}\tilde{\mathbf{y}}$, $\mathbf{X}^* = \mathbf{W}^{1/2}\mathbf{X}$ and $\boldsymbol{\epsilon}^* = \mathbf{W}^{1/2}\boldsymbol{\epsilon}$, the linear model becomes:

$$\mathbf{y}^* = \mathbf{X}^*\boldsymbol{\beta} + \boldsymbol{\epsilon}^* \tag{5}$$

where $\boldsymbol{\epsilon^*} \sim N(0, \sigma^2)$.

The score statistic can then be equivalently written as

$$Q(\tilde{\boldsymbol{\beta}}, \hat{\rho}) = \frac{1}{\hat{\sigma}^2}(\tilde{\mathbf{y}}^* - \tilde{\boldsymbol{\mu}}^*)^T \mathbf{D}^{1/2}\mathbf{K}_{\hat{\rho}}\mathbf{D}^{1/2}(\tilde{\mathbf{y}}^* - \widehat{\boldsymbol{\mu}}^*) \tag{6}$$

Following Chen's results (Chen et al., 2016), the approximate form of Equation (6) is

$$Q(\tilde{\boldsymbol{\beta}}, \hat{\rho}) \propto \frac{\boldsymbol{\epsilon}^{*T}\mathbf{P}_0\mathbf{D}^{1/2}\mathbf{K}_{\hat{\rho}}\mathbf{D}^{1/2}\mathbf{P}_0\boldsymbol{\epsilon}^*}{\boldsymbol{\epsilon}^{*T}\mathbf{P}_0\boldsymbol{\epsilon}^*} \triangleq \mathbf{R} \tag{7}$$

where

$$\begin{aligned}\mathbf{P}_0 &= \mathbf{I} - \mathbf{X}^*(\mathbf{X}^{*T}\mathbf{X}^*)^{-1}\mathbf{X}^{*T} \\ &= \mathbf{I} - \tilde{\mathbf{W}}^{\frac{1}{2}}\mathbf{X}(\mathbf{X}^T\tilde{\mathbf{W}}\mathbf{X})^{-1}\mathbf{X}^T\tilde{\mathbf{W}}^{\frac{1}{2}}.\end{aligned}$$

Assuming the normality of $\boldsymbol{\epsilon}^*$, the p value can be calculated using Davies method based on $\mathbf{r}$, i.e., the observed score statistic of $\mathbf{R}$:

$$\Pr(\mathbf{R} \leq \mathbf{r}) = \Pr\left(\frac{\boldsymbol{\epsilon}^{*T}(\mathbf{P}_0\mathbf{D}^{1/2}\mathbf{K}_{\hat{\rho}}\mathbf{D}^{1/2}\mathbf{P}_0 - r\mathbf{P}_0)\boldsymbol{\epsilon}^*}{\sigma^2} \leq 0\right) \tag{8}$$

where $\mathbf{r}$ is the observed score statistic of $\mathbf{R}$ and $Pr()$ denote the probability function.

Definition 1. Conditionally Negative Definite (CND)

A symmetric function $h : \chi \times \chi \to \mathbb{R}$, such that, for all $n \in \mathbb{N}$, $c_i \in \mathbb{R}$ and for all $z_i \in \chi$:

$$\sum_{i,j=1}^{n} c_i c_j h(z_i, z_j) \leq 0, \tag{9}$$

if the condition $\sum_{i=1}^{n} c_i = 0$ holds, is called a conditionally negative definite (CND) function.

1.1 Embedding of Conditionally Negative Definite (CND) via Hilbert Space

In the previous section, we discussed the square of the JS divergence is a CND distance metric. (Schölkopf, 2000; Schölkopf et al., 2002) explained that conditionally positive definite (CPD) kernels are 'as good' as positive definite (PD) kernels when used in translation invariant algorithms such as SVM, kernel PCA or logistic kernels. (Liu et al., 2021).

Remark 1. A Conditionally Positive Definite (CPD) $k(\cdot, \cdot)$ can be obtained from a distance $d(\cdot, \cdot)$ by defining $k(z_i, z_j) = -\frac{1}{2}d(z_i, z_j)$ for all $z_i,\ z_j \in \chi$.

Proposition 1. *Let $d : \chi \times \chi \to \mathbb{R}$ be a distance function between two elements of a set χ. There is a Hilbert space embedding of $(\chi, d(\cdot,\cdot))$ if and only if $d(\cdot,\cdot)^2$ is a conditionally negative function (Schoenberg, 1938).*

(Schoenberg, 1938) stated that a distance $d(\cdot,\cdot)$ defines a embedding of the points into a Hilbert space if and only if $d(\cdot,\cdot)^2$ is a CND. The distance between any pair of points z_i, z_j in the embedding is exactly the distance $d(z_i, z_j)$. In this extension to cytoKernel for single cell distributions, we propose a general technique based on the multidimesional scaling theory (MDS) to compute a positive definite (PD) kernel from a conditionally negative definite (CND) squared distance. We replace $d(z_i, z_j)$ in (**??**) with $\sqrt{D_{JS}(P_1, P_2)}$ to include the full distribution assumptions of single cell data for pairwise subjects.

Derivation of a kernel matrix from a CND distance

We construct a positive definite (PD) kernel matrix K for a finite set of points $n \in \mathbb{N}$ points given their CND distance matrix. We provided an interpretation of this kernel in the embedding (Supplementary materials). Let $\{z_1, \dots, z_n\}$ be a set of $n \in \mathbb{N}$ points lying in the input space χ and A denote an $n \times n$ matrix with $a_{ij} = -\frac{1}{2}d(z_i, z_j)^2$. The kernel matrix K is defined as

$$K = HAH, \tag{10}$$

where $H = I - \frac{E}{n}$, such that, I is an $n \times n$ identity matrix and E is an $n \times n$ matrix with each element being equal to 1. H is called the projection matrix and is also referred to as the centering matrix (Martin and Maes, 1979; Mardia and Riley, 2021). It projects the vectors into a $n-1$ dimensional space orthogonal to the column vector 1, which means that $(Hl)^T 1 = 0$ for all $l \in \mathbb{R}^n$. Now, we will show that K is PD.

Proposition 2. *Let $d : \chi \times \chi \to \mathbb{R}$ be a distance function between two probability distributions of a input space χ and $z_1, \dots, z_n \in \chi$. Let A denote a $n \times n$ matrix with $a_{ij} = -\frac{1}{2}d(z_i, z_j)^2$ and let H denote a $n \times n$ matrix with $a_{ij} = -\frac{1}{2}d(z_i, z_j)^2$ and let H denote a $n \times n$ matrix defined as $H = I - \frac{E}{n}$. Then, the matrix $K = HAH$ is PD if $d(\cdot,\cdot)^2$ is CND.*

Proof. *If $d(\cdot,\cdot)$ is CND, the following inequalities hold*

$$\begin{aligned} & -2(Hl)^T A(Hl) \leq 0 \quad \forall z \in \mathbb{R}^n \\ \iff \quad & l^T(HAH)l \geq 0 \quad \forall z \in \mathbb{R}^n. \end{aligned} \tag{11}$$

Equation (11) explicitly defines $K = HAH$ as a PD matrix.

Mercer theorem (Mercer, 1909): K is a dot product matrix for a set of n points embedded into a Hilbert space: $k_{ij} = \langle \phi(z_i), \phi(z_j) \rangle$ for all $1 \leq i, j \leq n$. Next, we show that the distance between two points z_i, z_j in the embedding induced by K is $d(z_i, z_j)$.

Proposition 3. *Let K be a PD matrix defined as $K = HAH$ (Equation (10)). Then the distance between two points z_i, z_j in the embedding induced by K in $d(x_i, x_j)$.* **Proof.** *The squared distance between two points z_i and z_j with $1 \leq i, j \leq n$ in the embedding wih respect to K is*

$$
\begin{aligned}
||\phi(z_i) - \phi(z_j)||^2 &= k_{ii} + k_{jj} - 2k_{ij} \\
&= (e_i - e_j)^T HAH(e_i - e_j) \\
&= (e_i - e_j)^T \left(I - \frac{E}{n}\right) A \left(I - \frac{E}{n}\right) (e_i - e_j) \\
&= (e_i - e_j)^T A(e_i - e_j) \\
&= -2a_{ij},
\end{aligned}
$$

where e_i is an $n \times 1$ vector with all elements being equal to 0 except for the ith being equal to 1.

According to Schoenberg theorem (Schoenberg, 1938), any distance $d(\cdot, \cdot)$, more specifically in our case, the JS divergence with a squared CND, induces an embedding in a Hilbert space. We presented a proof of a finite set of points by defining a PD kernel matrix K and also by showing that the distance in the embedding induced by K is $d(\cdot, \cdot)$.

1.1.1 Rationale behind the JSD embedding induced by the kernel

We explored a kernel interpretation derived from a CND distance matrix, as outlined in Equation (10). Our focus was on the computation of the relative dot product matrix within the embedding defined by K. This process involves a method similar to that used in MDS (Cox, 2001; Mardia and Riley, 2021) for deriving a dot product matrix from a CND distance matrix, modified for application in Hilbert space. Given that a CND distance $d(\cdot, \cdot)$ facilitates a Hilbert space embedding, the following relationship is established:

$$
\begin{aligned}
d(z_i, z_j)^2 &= ||\phi(z_i) - \phi(z_j)||^2 \\
&= ||\phi(z_i)||^2 + ||\phi(z_j)||^2 - 2\langle \phi(z_i), \phi(z_j) \rangle
\end{aligned}
\tag{12}
$$

While the distance remains invariant to translation, the dot product does not share this property. To address this, we position the origin of $\mathcal{H}_k$ at the centroid of n points. This adjustment permits the use of the translated mapping $\psi : \chi \to \mathcal{H}_k$, defined as $\psi(z) = \phi(z) - \frac{1}{n} \sum_{k=1}^{n} \phi(z_k)$. This centering method aligns well with the JS divergence, which is relative to the mean of probability distributions. We demonstrate that in the original embedding, the kernel performs the computation as follows:

$$\langle \psi(z_i), \psi(z_j) \rangle = \langle \phi(z_i), \phi(z_j) \rangle - \frac{1}{n} \langle \phi(z_i), \sum_{k=1}^{n} \phi(z_k) \rangle$$

$$= \frac{1}{n} \langle \phi(z_j), \sum_{k=1}^{n} \phi(z_k) \rangle + \frac{1}{n^2} \langle \sum_{k=1}^{n} \phi(z_k), \sum_{k=1}^{n} \phi(z_k) \rangle \tag{13}$$

Summarizing over index i and indices i, j, and given that $\sum_{k=1}^{n} \psi(z_k) = 0$ in Equation (12) (applicable for any $\psi(\cdot)$), we derive the following identities:

Identity 1.

$$||\phi(z_i)||^2 = \frac{1}{n} \sum_{k=1}^{n} \langle \psi(z_k), \psi(z_k) \rangle - \frac{1}{n} \sum_{k=1}^{n} ||\phi(z_i) - \phi(z_k)||^2 \tag{14}$$

Identity 2.

$$\frac{2}{n} \sum_{k=1}^{n} \langle \psi(z_k), \psi(z_k) \rangle = \frac{1}{n^2} \sum_{k,l=1}^{n} ||\phi(z_k) - \phi(z_l)||^2 \tag{15}$$

Substituting these identities into Equation (12), we can express the dot product as follows:

$$\langle \psi(z_i), \psi(z_j) \rangle = -\frac{1}{2} (||\phi(z_i) - \phi(z_j)||^2$$

$$= \frac{1}{n} \sum_{k=1}^{n} ||\phi(z_i) - \phi(z_k)||^2 + \frac{1}{n} \sum_{k=1}^{n} ||\phi(z_j) - \phi(z_k)||^2$$

$$- \frac{2}{n^2} \sum_{k,l=1}^{n} ||\phi(z_k) - \phi(z_l)||^2) \tag{16}$$

It is pertinent to note that the dot product in Hilbert space is interpretable as distances between points. Owing to the translation invariance of the distance, we have:

$$||\psi(z_i) - \psi(z_j)||^2 = ||\phi(z_i) - \phi(z_j)||^2 \tag{17}$$

Moreover, Equation (16) can be reformulated into a kernel matrix form $K = HAH$ as defined in Equation (10).

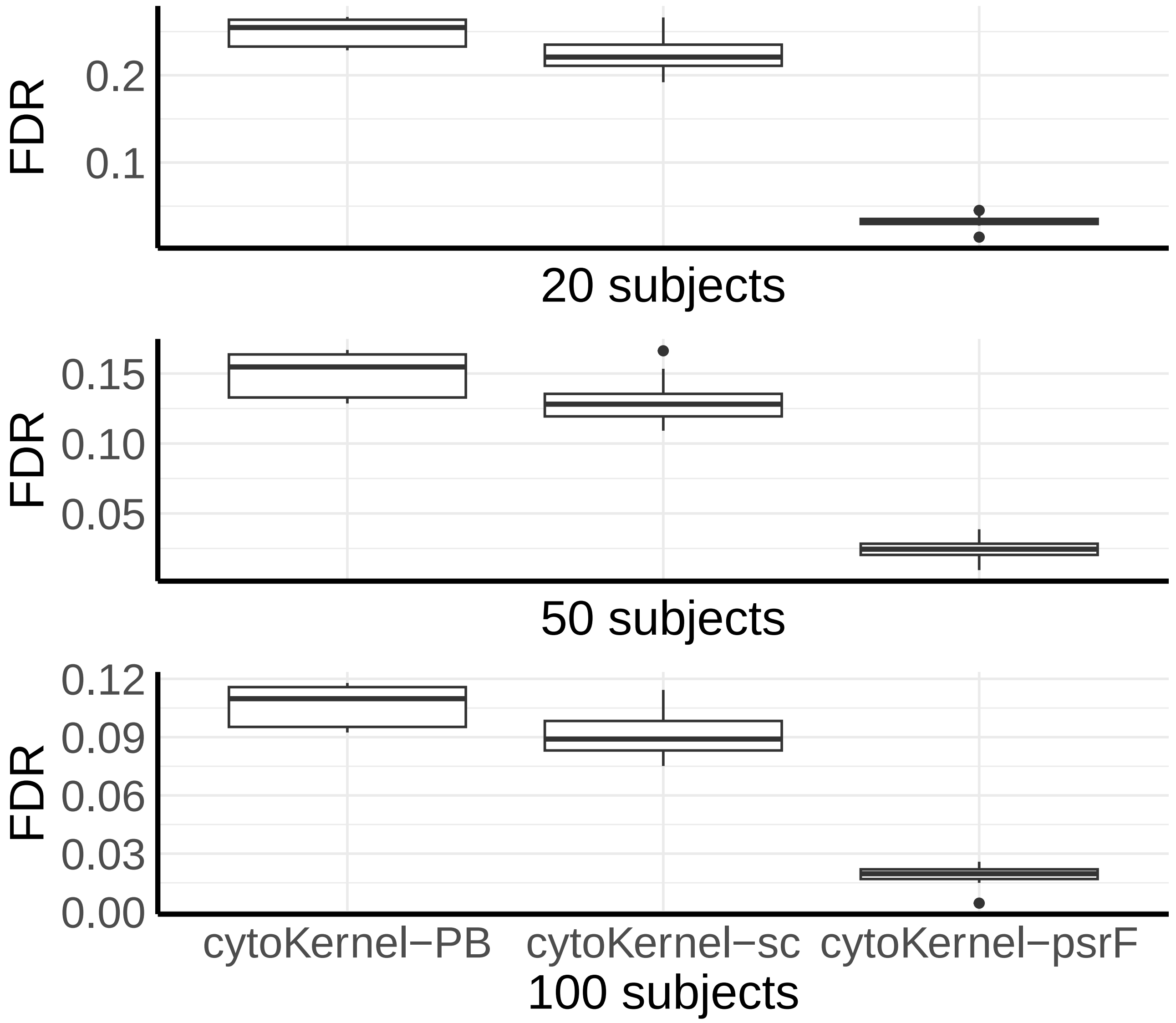

Figure 1: FDR plots for splatPop simulations based on 10 simulated datasets by varying sample sizes.

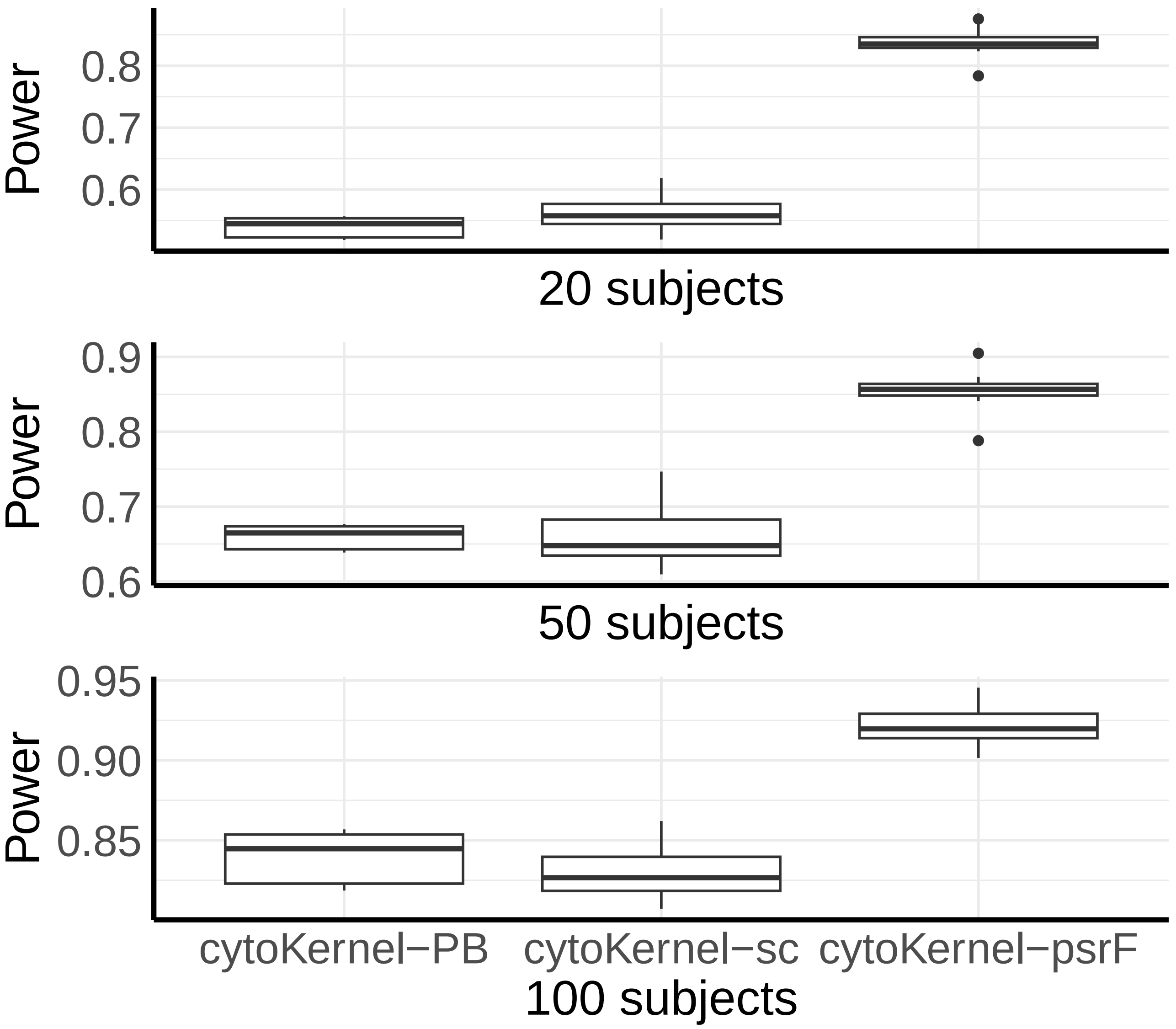

Figure 2: Discriminative Power plots for splatPop simulations based on 10 simulated datasets by varying sample sizes.

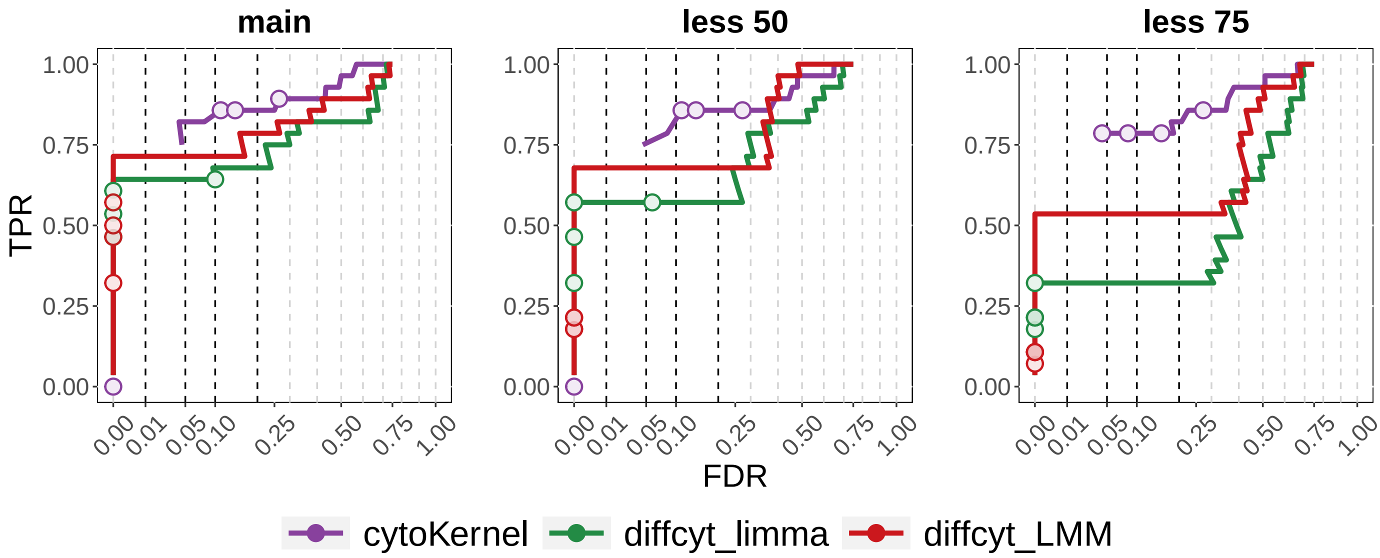

Figure 3: cytoKernel displays high power while controlling for false positive and false discovery rates, TPR versus FDR in diffcyt semisimulated data. "main", "less 50", and "less 75" indicate the main simulation, and those where differential effects are diluted by 50 and 75%, respectively. Each simulation consists of 88, 435 cells and two groups of eight samples each. Circles indicate observed FDR for 0.01, 0.05, 0.1, and 0.2 level of significance thresholds. Cells were clustered into eight populations based on manually annotated cell types as in the distinct simulation design (Crowell et al., 2020; Weber et al., 2019; Tiberi et al., 2022).

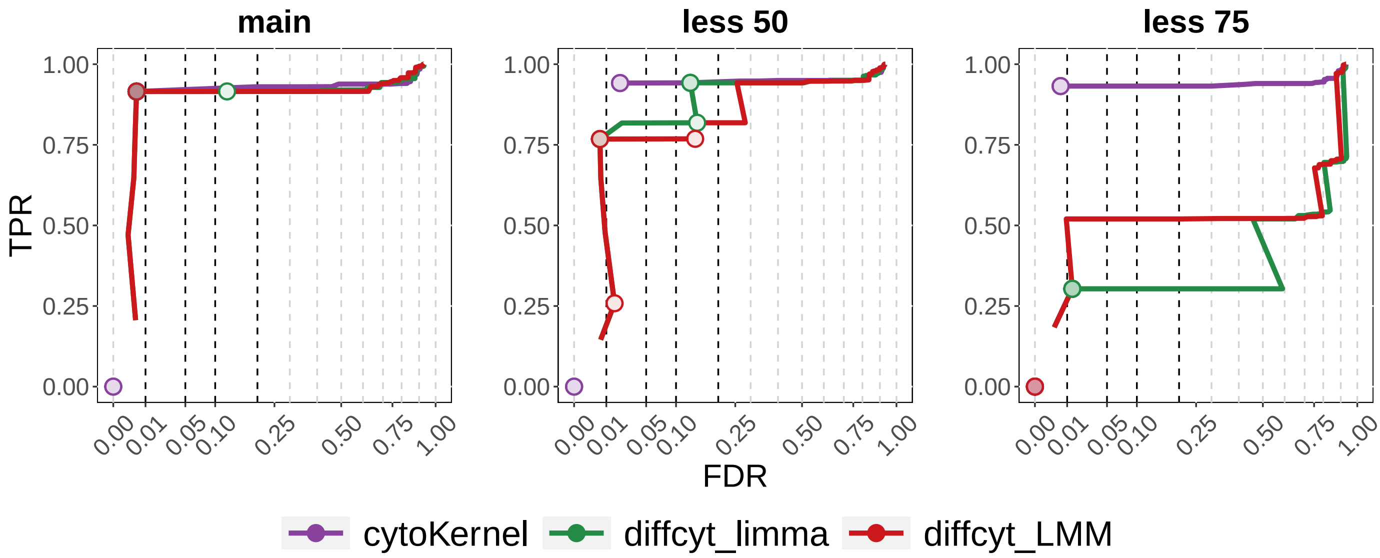

Figure 4: cytoKernel displays high power while controlling for false positive and false discovery rates, TPR versus FDR in diffcyt semisimulated data. “main”, “less 50“, and “less 75” indicate the main simulation, and those where differential effects are diluted by 50 and 75%, respectively. Each simulation consists of 88, 435 cells and two groups of eight samples each. Circles indicate observed FDR for 0.01, 0.05, 0.1, and 0.2 level of significance thresholds. Cells were grouped in 100 high-resolution clusters via unsupervised clustering as in the distinct simulation design (Crowell et al., 2020; Weber et al., 2019; Tiberi et al., 2022).

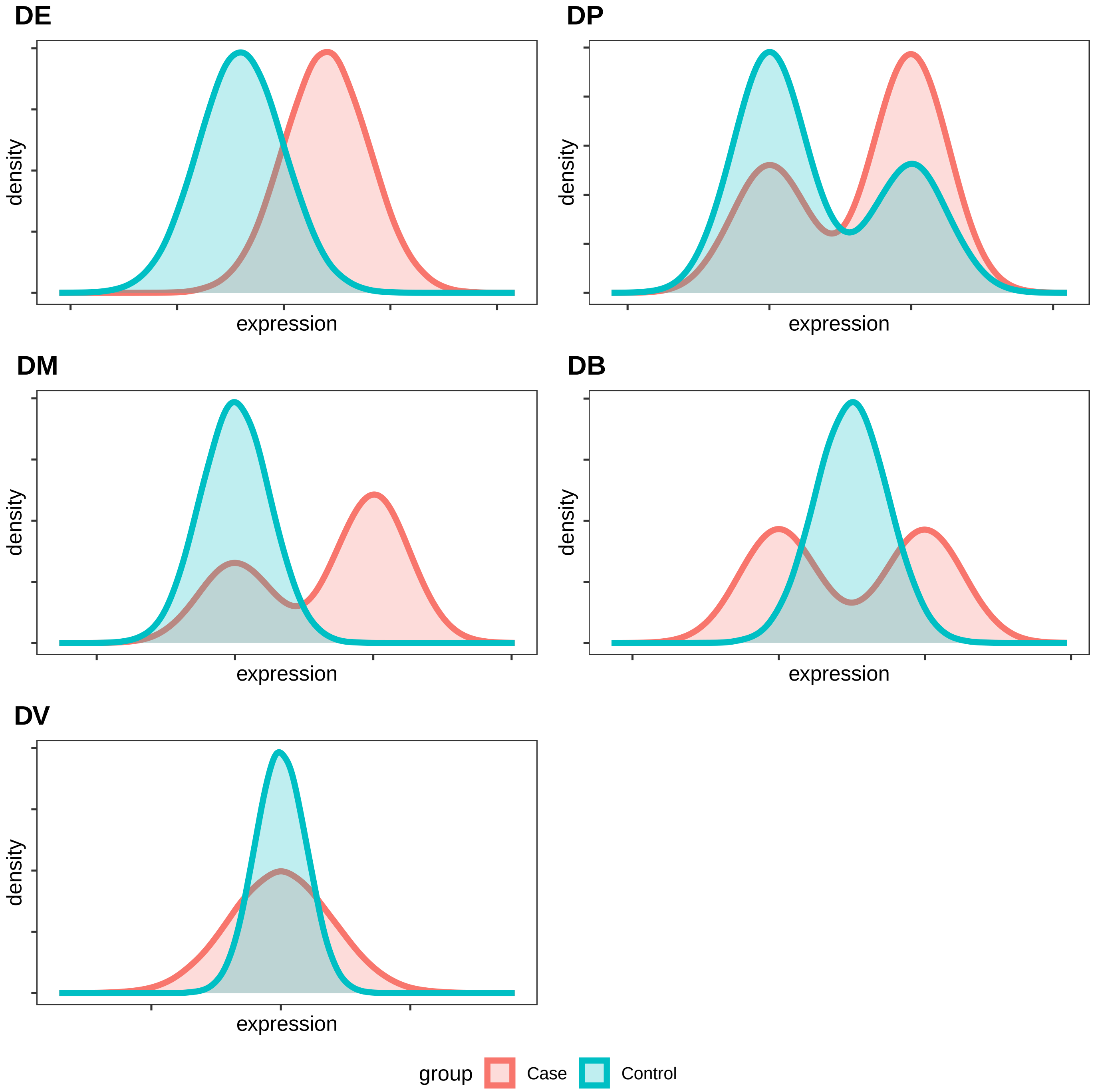

Figure 5: Density of five distinct differential patterns. These include Differential Variability (DV) proposed by Tiberi et al. (2022) and four patterns outlined by Korthauer et al. (2016): Differential Expression (DE), Differential Proportion (DP), Differential Modality (DM), and a combination pattern of Differential Modality with Different Component Means (DB).

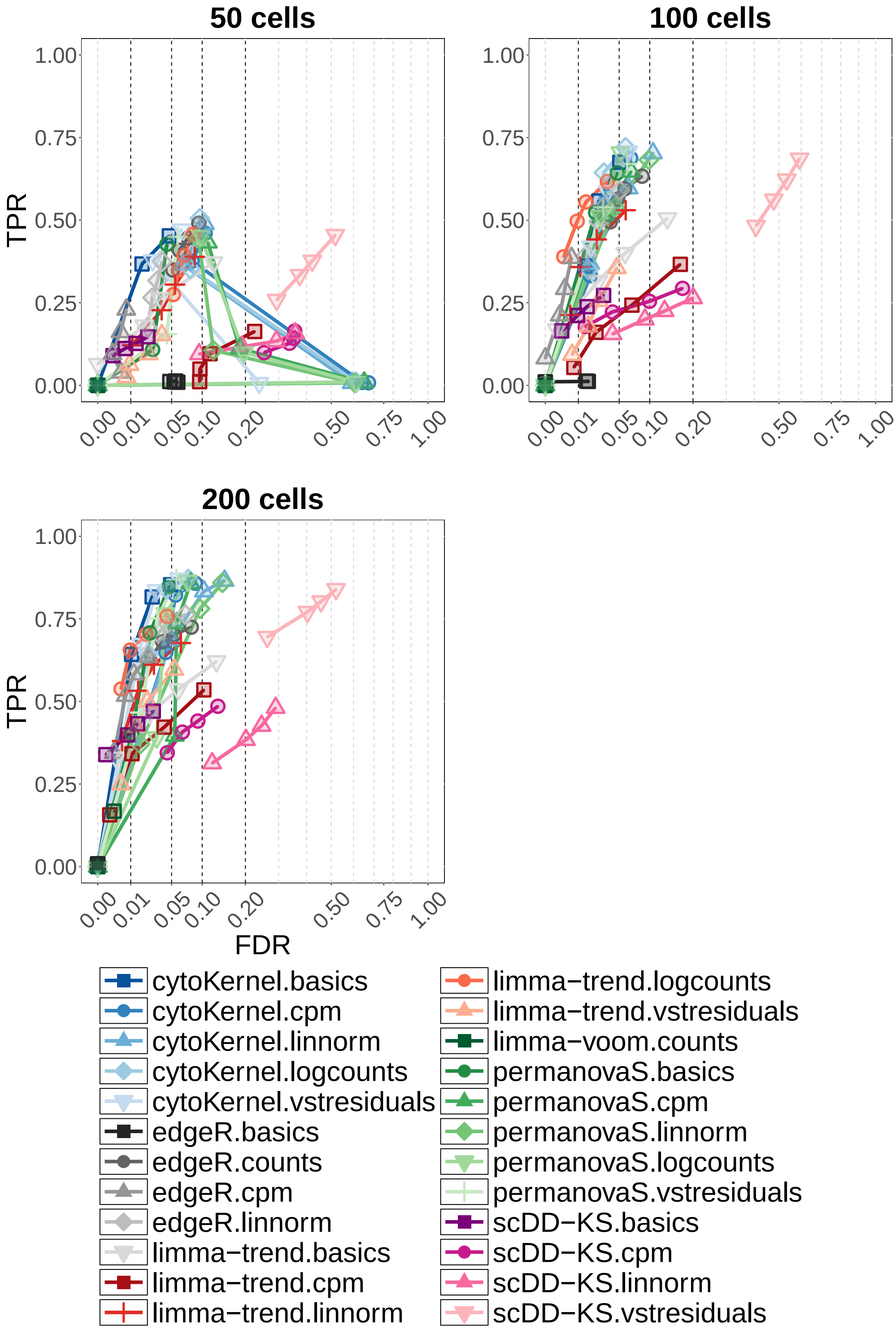

Figure 6: cytoKernel demonstrates better performance when varying the number of available cells. TPR versus FDR in muscat simulated data; with 50, 100, 200 and 400 cells per cluster-sample combination, corresponding to a total of 900, 1800, 3600, and 7200 cells, respectively. Results are aggregated over the five replicate simulations of each differential type (DE, DP, DM, DB, and DV), contributing in equal fraction. Each individual simulation replicate consists of 4000 genes, three cell clusters and two groups of three samples each. Circles indicate observed FDR for 0.01, 0.05, 0.1, and 0.2 significance thresholds as in the distinct simulation design (Crowell et al., 2020; Tiberi et al., 2022).

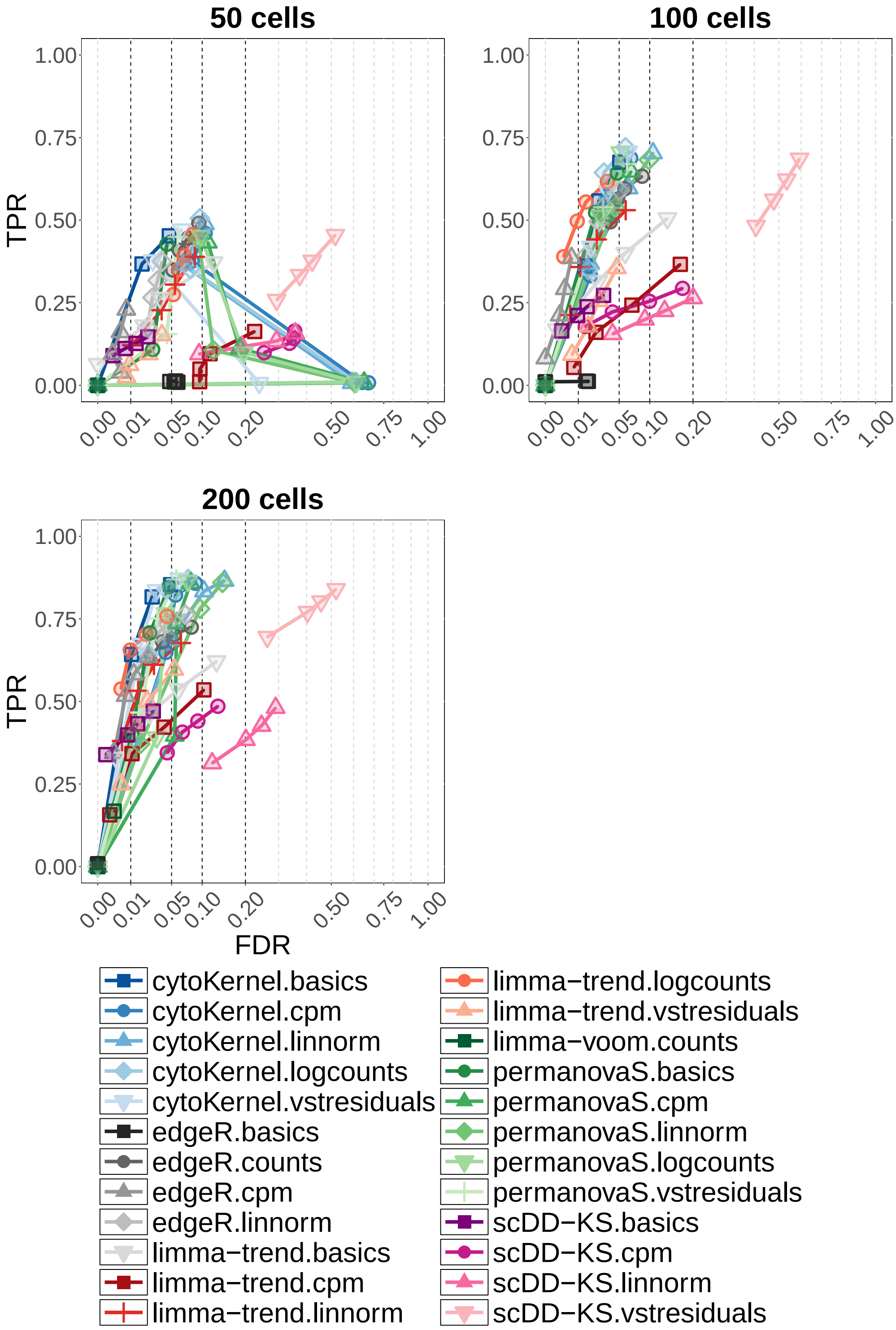

Figure 7: cytoKernel demonstrates better performance and effectively remains unchanged with batch-effects. TPR versus FDR in muscat simulated data; with 50, 100, 200 and 400 cells per cluster-sample combination, corresponding to a total of 900, 1800, 3600, and 7200 cells, respectively. Results are aggregated over the five replicate simulations of each differential type (DE, DP, DM, DB, and DV), contributing in equal fraction. Each individual simulation replicate consists of 4000 genes, three cell clusters and two groups of three samples each. Circles indicate observed FDR for 0.01, 0.05, 0.1, and 0.2 significance thresholds as in the distinct simulation design (Crowell et al., 2020; Tiberi et al., 2022).

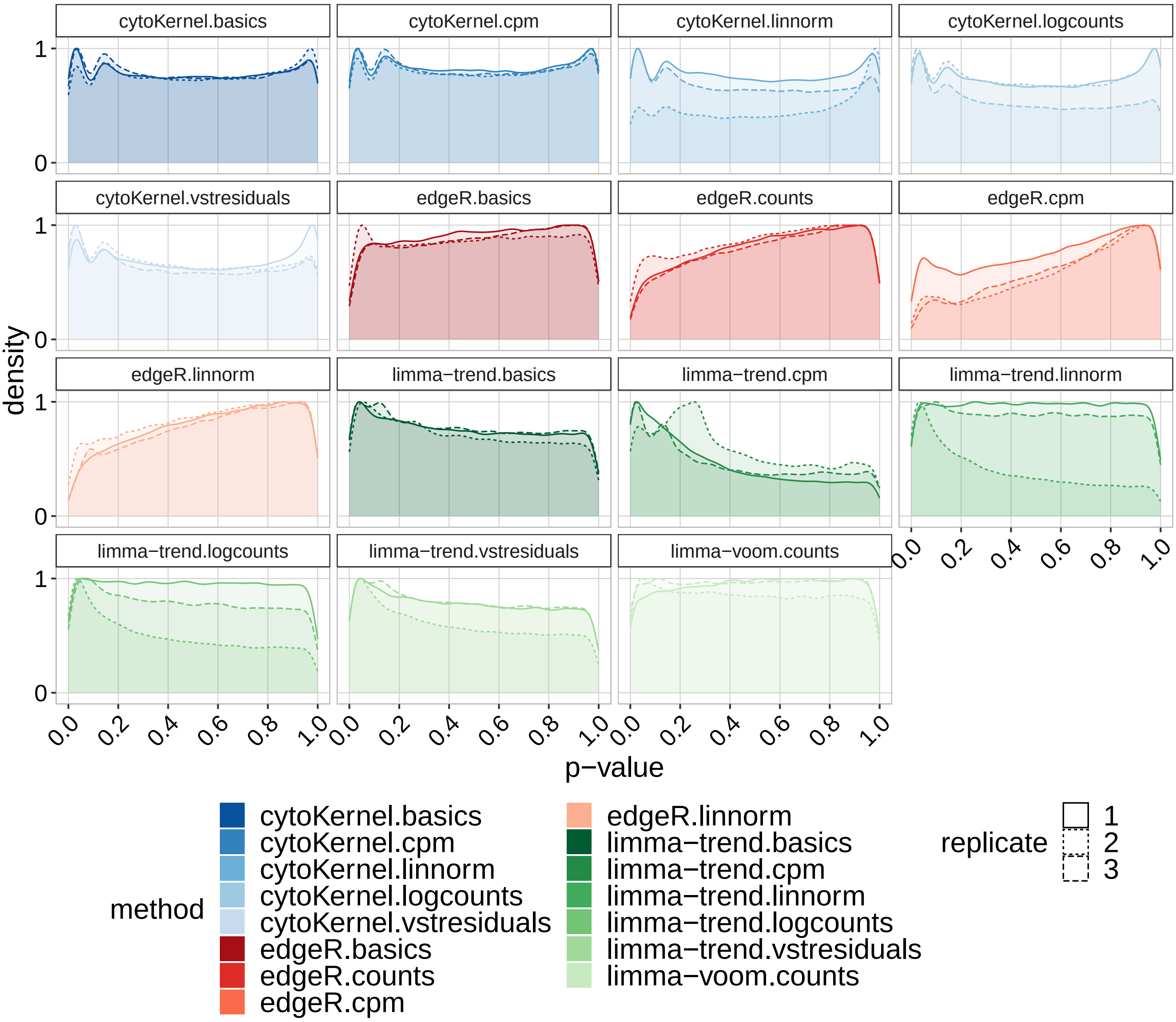

Figure 8: Density of raw p-values in null T-cells data, comprising 12 samples with 11,138 cells across 11 clusters. Each replicate in these datasets represents a random division of samples into two groups, highlighting the distribution of p-values obtained. The 'cytoKernel' method demonstrates an almost-uniform distribution of null p-values.

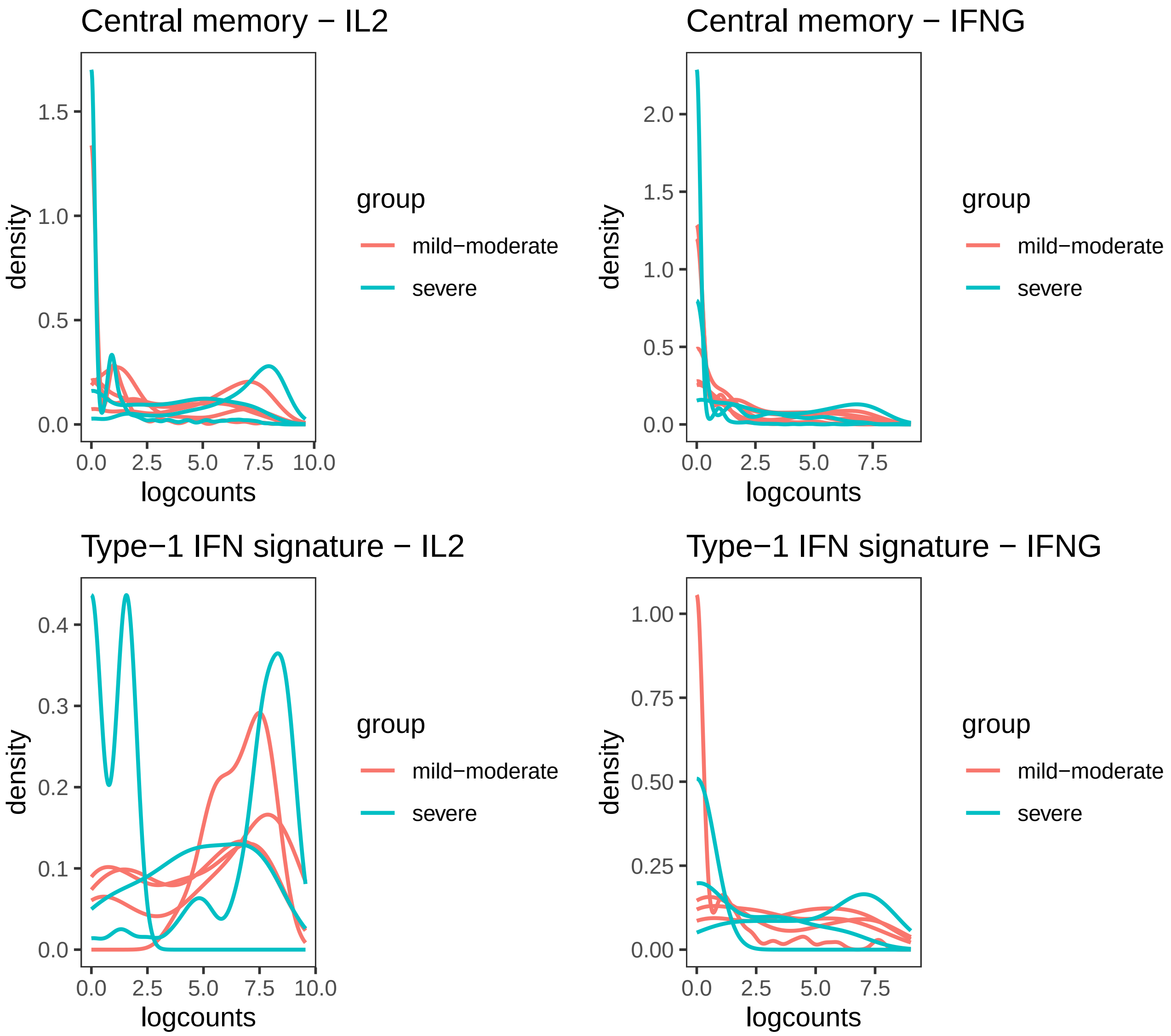

Figure 9: Empirical distributions of IL2 and IFNG (Bacher et al., 2020).

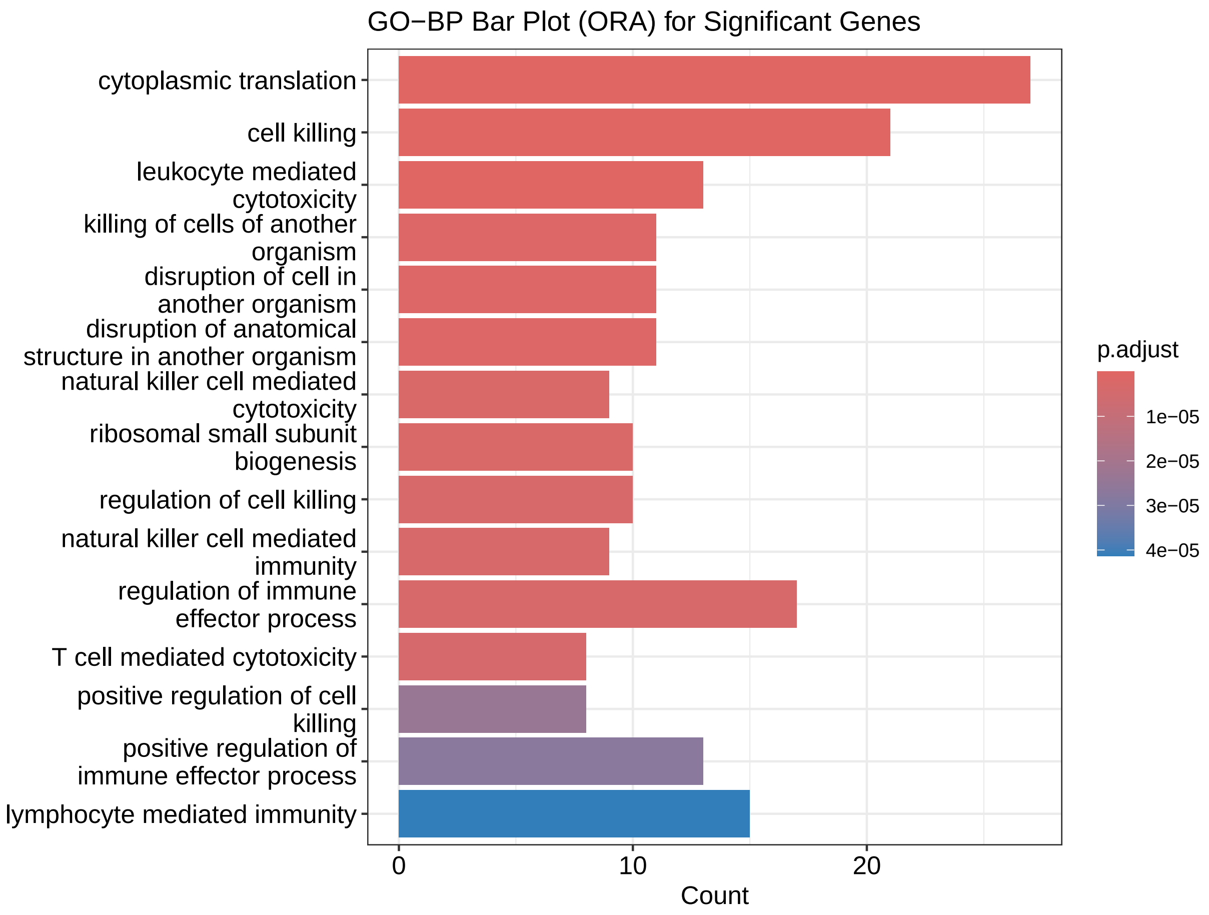

Figure 10: Barplot ranks significant pathways by enrichment score and highlights the most overrepresented terms. Over-representation analysis (ORA) for the "Cytotoxic / Th1" cluster, extracted from the mild, moderate, and severe COVID-19 dataset (Bacher et al., 2020). Genes significantly associated with this cluster ($adjusted.p < 0.1$) were mapped to Entrez IDs and analyzed using Gene Ontology Biological Process (GO-BP) terms.

Table 1: Computing time (in minutes) across differential expression patterns in muscat based simulations (Crowell et al., 2020; Tiberi et al., 2022). Times are averaged over five replicates with 3,600 cells (200 per cluster-sample combination).Estimated computing time (in minutes) when run on 8 cores. Methods not supporting parallelization:edgeR and limma.

**Method**	**DE**	**DP**	**DM**	**DB**	**DV**	**null**	**avg**
scDD-perm.vstresiduals	821.9	781.2	770.0	612.0	841.5	865.9	782.1
scDD-perm.linnorm	222.5	215.6	219.0	248.6	220.1	207.1	222.6
scDD-perm.cpm	199.3	203.0	205.8	221.4	202.2	192.3	204.3
scDD-perm.basics	200.5	203.2	207.5	217.5	204.4	192.4	204.3
permanovaS.vstresiduals	10.5	9.5	13.2	9.6	7.2	1.8	8.6
permanovaS.logcounts	12.0	8.5	10.2	7.2	5.6	3.0	7.8
permanovaS.cpm	8.0	10.2	11.9	6.6	4.2	2.4	7.2
permanovaS.linnorm	10.8	7.2	11.9	5.4	3.2	3.6	7.0
permanovaS.basics	6.8	9.6	10.2	5.0	3.5	3.0	6.4
cytoKernel.vstresiduals	2.1	1.9	2.2	1.6	1.2	0.3	1.5
cytoKernel.logcounts	2.0	1.7	1.7	1.2	0.8	0.6	1.3
cytoKernel.cpm	2.0	1.7	1.7	1.1	0.7	0.6	1.3
cytoKernel.linnorm	1.8	1.6	1.7	1.2	0.8	0.6	1.3
cytoKernel.basics	1.7	1.6	1.7	1.0	0.7	0.6	1.2
scDD-KS.vstresiduals	0.7	0.7	0.7	0.9	0.6	0.6	0.7
scDD-KS.cpm	0.5	0.5	0.5	0.5	0.5	0.5	0.5
scDD-KS.linnorm	0.5	0.5	0.5	0.6	0.4	0.4	0.5
scDD-KS.basics	0.4	0.4	0.5	0.5	0.4	0.4	0.5
edgeR.cpm	0.2	0.2	0.2	0.2	0.2	0.2	0.2
edgeR.counts	0.2	0.2	0.2	0.2	0.2	0.1	0.2
edgeR.linnorm	0.2	0.2	0.2	0.2	0.2	0.2	0.2
edgeR.basics	0.1	0.1	0.1	0.1	0.1	0.1	0.1
limma-trend.cpm	0.1	0.1	0.1	0.1	0.1	0.1	0.1
limma-voom.counts	0.1	0.1	0.1	0.1	0.1	0.1	0.1
limma-trend.basics	0.1	0.1	0.1	0.1	0.1	0.1	0.1
limma-trend.linnorm	0.1	0.1	0.1	0.1	0.1	0.1	0.1
limma-trend.vstresiduals	0.1	0.1	0.1	0.1	0.1	0.1	0.1
limma-trend.logcounts	0.1	0.1	0.1	0.1	0.1	0.1	0.1

Method	0.1	0.05	0.01
cytoKernel.basics	0.12	0.08	0.02
cytoKernel.cpm	0.12	0.08	0.02
cytoKernel.linnorm	0.12	0.08	0.02
cytoKernel.logcounts	0.13	0.08	0.02
cytoKernel.vstresiduals	0.13	0.08	0.02
edgeR.basics	0.00	0.00	0.00
edgeR.counts	0.09	0.05	0.01
edgeR.cpm	0.05	0.02	0.00
edgeR.linnorm	0.08	0.04	0.01
limma-trend.basics	0.14	0.08	0.02
limma-trend.cpm	0.20	0.11	0.03
limma-trend.linnorm	0.15	0.08	0.02
limma-trend.logcounts	0.12	0.07	0.02
limma-trend.vstresiduals	0.13	0.07	0.01
limma-voom.counts	0.08	0.04	0.01

Table 2: Proportion of False Positives identified by each method at significance levels of 0.01, 0.05, and 0.1 in the Null Kang Lupus data.

Method	0.1	0.05	0.01
cytoKernel.basics	0.12	0.08	0.02
cytoKernel.cpm	0.11	0.07	0.02
cytoKernel.linnorm	0.12	0.08	0.02
cytoKernel.logcounts	0.14	0.10	0.03
cytoKernel.vstresiduals	0.14	0.09	0.03
edgeR.basics	0.09	0.04	0.01
edgeR.counts	0.06	0.03	0.00
edgeR.cpm	0.06	0.03	0.00
edgeR.linnorm	0.06	0.03	0.00
limma-trend.basics	0.14	0.08	0.02
limma-trend.cpm	0.19	0.12	0.05
limma-trend.linnorm	0.16	0.09	0.03
limma-trend.logcounts	0.15	0.08	0.02
limma-trend.vstresiduals	0.15	0.08	0.02
limma-voom.counts	0.11	0.06	0.02

Table 3: Proportion of False Positives identified by each method at significance levels of 0.01, 0.05, and 0.1 in the T cell data.

References

Bacher, P., E. Rosati, D. Esser, G. R. Martini, C. Saggau, E. Schiminsky, J. Dargvainiene, I. Schröder, I. Wieters, Y. Khodamoradi, et al. (2020). Low-avidity cd4+ t cell responses to sars-cov-2 in unexposed individuals and humans with severe covid-19. *Immunity 53*(6), 1258–1271.

Chen, H., M. C. Lau, M. T. Wong, E. W. Newell, M. Poidinger, and J. Chen (2016). Cytofkit: a bioconductor package for an integrated mass cytometry data analysis pipeline. *PLoS computational biology 12*(9), e1005112.

Chen, J., W. Chen, N. Zhao, M. C. Wu, and D. J. Schaid (2016). Small sample kernel association tests for human genetic and microbiome association studies. *Genetic epidemiology 40*(1), 5–19.

Cox, T. F. (2001). Multidimensional scaling used in multivariate statistical process control. *Journal of Applied Statistics 28*(3-4), 365–378.

Crowell, H. L., C. Soneson, P.-L. Germain, D. Calini, L. Collin, C. Raposo, D. Malhotra, and M. D. Robinson (2020). Muscat detects subpopulation-specific state transitions from multi-sample multi-condition single-cell transcriptomics data. *Nature communications 11*(1), 1–12.

Davies, R. B. (1980). Algorithm as 155: The distribution of a linear combination of χ 2 random variables. *Applied Statistics*, 323–333.

Davies, R. B. (1987). Hypothesis testing when a nuisance parameter is present only under the alternative. *Biometrika 74*(1), 33–43.

Duchesne, P. and P. L. De Micheaux (2010). Computing the distribution of quadratic forms: Further comparisons between the liu–tang–zhang approximation and exact methods. *Computational Statistics & Data Analysis 54*(4), 858–862.

Hastie, T. J. and D. Pregibon (2017). Generalized linear models. In *Statistical models in S*, pp. 195–247. Routledge.

Korthauer, K. D., L.-F. Chu, M. A. Newton, Y. Li, J. Thomson, R. Stewart, and C. Kendziorski (2016). A statistical approach for identifying differential distributions in single-cell rna-seq experiments. *Genome biology 17*(1), 1–15.

Lee, S., M. C. Wu, and X. Lin (2012). Optimal tests for rare variant effects in sequencing association studies. *Biostatistics 13*(4), 762–775.

Liu, F., X. Huang, Y. Chen, and J. A. Suykens (2021). Random features for kernel approximation: A survey on algorithms, theory, and beyond. *IEEE Transactions on Pattern Analysis and Machine Intelligence 44*(10), 7128–7148.

Mardia, K. V. and A. D. Riley (2021). The classical multidimensional scaling revisited. *arXiv preprint arXiv:2112.14503*.

Martin, N. and H. Maes (1979). *Multivariate analysis.* Academic Press London.

Mercer, J. (1909). Xvi. functions of positive and negative type, and their connection the theory of integral equations. *Philosophical transactions of the royal society of London. Series A, containing papers of a mathematical or physical character 209*(441-458), 415–446.

Schoenberg, I. J. (1938). Metric spaces and completely monotone functions. *Annals of Mathematics*, 811–841.

Schölkopf, B. (2000). The kernel trick for distances. *Advances in neural information processing systems 13.*

Schölkopf, B., A. J. Smola, F. Bach, et al. (2002). *Learning with kernels: support vector machines, regularization, optimization, and beyond.* MIT press.

Tiberi, S., H. L. Crowell, P. Samartsidis, L. M. Weber, and M. D. Robinson (2022). distinct: a novel approach to differential distribution analyses. *bioRxiv*, 2020–11.

Weber, L. M., M. Nowicka, C. Soneson, and M. D. Robinson (2019). diffcyt: Differential discovery in high-dimensional cytometry via high-resolution clustering. *Communications biology 2*(1), 1–11.
